# Well-posedness for a stochastic 2D Euler equation with transport noise

**DOI:** 10.1007/s40072-021-00233-7

**Published:** 2022-01-29

**Authors:** Oana Lang, Dan Crisan

**Affiliations:** Department of Mathematics, Imperial College London, London, SW7 2AZ UK

**Keywords:** Euler equation, Incompressible fluids, Stochastic fluid equations, Transport noise, 60H15, 60H30, 35R15, 35R60

## Abstract

We prove the existence of a unique global strong solution for a stochastic two-dimensional Euler vorticity equation for incompressible flows with noise of transport type. In particular, we show that the initial smoothness of the solution is preserved. The arguments are based on approximating the solution of the Euler equation with a family of viscous solutions which is proved to be relatively compact using a tightness criterion by Kurtz.

## Introduction

Consider the two-dimensional Euler equation modelling an incompressible flow perturbed by transport type stochasticity
with initial condition $\omega _{0}$, where $(\xi _{i})_i$ are time-independent divergence-free vector fields, the operator  is given by , and $(W^{i})_{i \in {\mathbb {N}}}$ is a sequence of independent Brownian motions. Classically, $u_{t}$ stands for the velocity of an incompressible fluid and $\omega _{t}=curl\ u_{t}$ is the corresponding fluid vorticity. The stochastic part considered here follows the Stochastic Advection by Lie Transport (SALT) theory (see [15, 18, 27, 33]) and corresponds to a stochastic integral of Stratonovich type.

The Euler equation is used to model the motion of an incompressible inviscid fluid. A representative aspect in this context is the study of the fluid vortex dynamics modelled by the vorticity equation. There is a vast literature on well-posedness in the deterministic setting, see e.g. [4, 10, 24, 35, 38, 39, 44, 51], and references therein.

The introduction of stochasticity into ideal fluid dynamics has received special attention over the past two decades. On one hand, comprehensive physical models can be obtained when the stochastic term accounts for physical uncertainties [15, 16, 18, 33], whilst, in some cases, the regularity properties of the deterministic solution can be improved when the right type of stochasticity is added [21, 26, 27, 31]. Global existence of smooth solutions for the stochastic Euler equation with multiplicative noise in both 2D and 3D has been obtained in [31]. In [8], a weak solution of the Euler equation with additive noise is constructed as an inviscid limit of the stochastic damped 2D Navier-Stokes equations. A martingale solution constructed also as a limit of Navier-Stokes equations but with cylindrical noise can be found in [13]. Existence and uniqueness results with different variations in terms of stochastic forcing and approximations can be found in [6, 19, 42, 45, 46] and references therein. An overview of results on this topic is provided in [7].

The analysis of nonlinear stochastic partial differential equations with noise of transport type has recently expanded substantially, see e.g., [2, 3, 5, 15, 16, 18, 30, 33]. Existence of a solution for the two-dimensional stochastic Euler equation with noise of transport type has been considered in [12]. While in [12] the authors prove the existence and pathwise uniqueness of a distributional solution in $L^{\infty }( {\mathbb {T}}^2)$, in this paper we are concerned with the existence of a strong solution and give conditions under which the solution enjoys smoothness properties.^1^ In [25], a random point vortices system is used to construct a so-called $\rho$-white noise solution. Local well-posedness and a Beale-Kato-Majda blow-up criterion for the three-dimensional case in the space ${\mathcal {W}}^{2,2}({\mathbb {T}}^3)$ has been obtained in [18]. Full well-posedness for a point vortex dynamics system based on this equation has been proven in [28]. The linear case has been considered in [26] and in [29].

In the sequel, ${\mathbb {T}}^2$ is the two-dimensional torus, $k\ge 2$ is a fixed positive integer, and ${\mathcal {W}}^{k,2}({\mathbb {T}}^2)$ is the usual Sobolev space (see Sect. 2). The main result of this paper is the following:

Theorem: Under certain conditions on the vector fields $(\xi _i)_i$ the two-dimensional stochastic Euler vorticity equation

1 $$\begin{aligned} d{\omega }_{t}+u_{t}\cdot \nabla {\omega }_{t}dt+\sum _{i=1}^{\infty }(\xi _{i}\cdot \nabla {\omega }_{t})\circ dW_{t}^{i}=0,\ \ \ \omega _{0}\in {\mathcal {W}}^{k,2}({\mathbb {T}}^{2}), \ \ \ \end{aligned}$$

admits a unique global (in time) solution which belongs to the space ${\mathcal {W}}^{k,2}({\mathbb {T}}^{2})$. Moreover, $\omega _t$ is a continuous function of the initial condition.

### Remark 1

As stated above, the stochastic terms in (1) are stochastic integrals of Stratonovich type. We interpret Eq. (1) in its corresponding Itô form, that is

2 $$\begin{aligned} d\omega _t + u_t \cdot \nabla \omega _tdt + \displaystyle \sum _{i=1}^{\infty } \xi _i \cdot \nabla \omega _tdW_t^i = \frac{1}{2}\displaystyle \sum _{i=1}^{\infty } \xi _i \cdot \nabla \big (\xi _i \cdot \nabla \omega _t\big )dt. \end{aligned}$$

The assumptions on the vector fields $(\xi _i)_i$ are described in Sect. 2. In short, they are assumed to be sufficiently smooth, their corresponding norms to decay sufficiently fast as *i* increases, so that the infinite sums in (1), respectively, in (2) make sense in the right spaces (see condition (4) below). Importantly, we do not require the additional assumption^2^

3 $$\begin{aligned} \sum _{i=1}^{\infty } \xi _i(x)\xi _i^{\star }(x) = cI_2 \ \ \end{aligned}$$

used in [12]. As a result, in the Itô version (2) of the SPDE, the term $\frac{1}{2}\displaystyle \sum \nolimits _{i=1}^{\infty } \xi _i \cdot \nabla (\xi _i \cdot \nabla \omega _t)$ does not reduce to $c\Delta \omega _t$. This would simplify the analysis as, in this case, the Laplacian commutes with higher order derivatives. Morever, it commutes with the operation of convolution with the Biot–Savart kernel, an essential ingredient used in [12]. The general term $\frac{1}{2}\displaystyle \sum \nolimits _{i=1}^{\infty } \xi _i \cdot \nabla (\xi _i \cdot \nabla \omega _t)$ makes the analysis harder. We succeed in controlling it by considering it in tandem with the term $\displaystyle \sum \nolimits _{i=1}^{\infty }\displaystyle \int \nolimits _{{\mathbb {T}}^2} (\xi _i \cdot \nabla \omega _t)^2dx$ coming from the quadratic variation of the stochastic integrals (see Lemma 25i.) appearing in the evolution equation for the process $t\mapsto \Vert \omega _t\Vert ^2$. A similar technical difficulty appears when trying to control the high-order derivatives of the vorticity. Nonetheless, this is achieved through a set of inequalities (see Lemma 25) that have first been introduced in the literature by Krylov and Rozovskii [32, 36] and recently used by Crisan et al. [18]. Again, we emphasize that these rather surprising inequalities hold true without imposing assumptions on the driving vectors $(\xi _i)_i$ other than on their smoothness and summability. This finding is particularly important when using this model for the purpose of uncertainty quantification and data assimilation: for example, in [15–17], the driving vectors $(\xi _i)_i$ are *estimated* from data and not a priori chosen. The methodology used in these papers does not naturally lead to driving vector fields that satisfy Assumption (3) so removing it is essential for our research programme.

We emphasize that the appearance of the second order differential operator $\omega \mapsto \frac{1}{2}\sum _{i=1}^{\infty } \xi _i \cdot \nabla (\xi _i \cdot \nabla \omega )$ in the Itô version of the Euler equation does not give the equation a parabolic character, even if one assumes the restriction (3) with *c* chosen strictly positive. Equation (1) is truly a transport type equation and one cannot expect the initial condition to be smoothed out. The best scenario is to prove that the initial level of smoothness of the solution is preserved. This is indeed the main finding of our research. Moreover, we show that our result can be extended to cover also $L^{\infty }$-solutions in the Yudovich sense.

The paper is organised as follows: In Sect. 2 we introduce the main assumptions, key notations, and some preliminary results. In Sect. 3 we present our main results: in Sect. 3.1 we show that the solution is almost surely pathwise unique, while in Sect. 3.2 we prove existence of a strong solution (in the sense of Definition 3). In Sect. 4 we proceed with an extensive analysis of a truncated form of the Euler equation: uniqueness (Sect. 4.1) and existence - based on a new approximating sequence introduced in Sect. 4.2. At the end of this section we show continuity with respect to initial conditions for the original equation. In Sect. 5 we show existence, uniqueness, and continuity for the approximating sequence of solutions constructed in Sect. 4.2. In Sect. 6 we show that the family of approximating solutions is relatively compact. In Sect. 7 we present an extension of the main results to the Yudovich setting. The paper is concluded with an “Appendix” that incorporates a number of proofs of the technical lemmas and statements of some classical results.

## Preliminaries

We summarise the notation used throughout the paper. Let $(\Omega , {\mathcal {F}}, ({\mathcal {F}}_t)_t, {\mathbb {P}})$ be a filtered probability space, with the sequence $\left( W^{i}\right) _{i\in {\mathbb {N}}}$ of independent Brownian motions defined on it. Let *X* be a generic Banach space. Throughout the paper *C* is a generic notation for constants, whose values can change from line to line.

- We denote by ${\mathbb {T}}^{d}={\mathbb {R}}^{d}/{\mathbb {Z}}^{d}$ the d-dimensional torus. In our case $d=2$.
- Let $\alpha = (\alpha _1, \alpha _2, \ldots , \alpha _d) \in {\mathbb {N}}^d, \ d>0$, be a multi-index of length $|\alpha | = \displaystyle \sum \nolimits _{j=1}^{d}\alpha _j$. Then $\partial ^{\alpha } = \partial ^{(\alpha _1, \alpha _2, \ldots , \alpha _d)} = \partial _1^{\alpha _1}\partial _2^{\alpha _2} \ldots \partial _d^{\alpha _d}$ denotes the differential operator of order $|\alpha |$, with $\partial ^{(0,0, \ldots , 0)}f = f$ for any function *f* defined on ${\mathbb {T}}^d$, and $\partial _i^{\alpha _i} = \frac{\partial ^{\alpha _i}}{\partial x_i^{\alpha _i}}$, $x \in {\mathbb {T}}^d$. In our case $d=2$ and $|\alpha | \le k$.
- $L^{p} ({\mathbb {T}}^{2}; X)$^3^ is the class of all measurable *p*-integrable functions *f* defined on the two-dimensional torus, with values in *X* (*p* is a positive real number). The space is endowed with its canonical norm $\Vert f\Vert _p = \bigg (\displaystyle \int \nolimits _{{\mathbb {T}}^2} \Vert f\Vert _X^p dx\bigg )^{1/p}.$ Conventionally, for $p = \infty$ we denote by $L^{\infty }$ the space of essentially bounded measurable functions.
- For $a,b\in L^{2}\left( {\mathbb {T}}^{2}\right)$, we denote by $\langle \cdot ,\cdot \rangle$ the scalar product
  $$\begin{aligned} \langle a,b\rangle :=\displaystyle \int \limits _{{\mathbb {T}}^{2}}a(x)\cdot b(x)dx. \end{aligned}$$
- ${\mathcal {W}}^{m,p}({\mathbb {T}}^{2})$ is the Sobolev space of functions $f \in L^p({\mathbb {T}}^2)$ such that $D^{\alpha }f \in L^p({\mathbb {T}}^2)$ for $0 \le |\alpha | \le m$, where $D^{\alpha }f$ is the distributional derivative of *f*. The canonical norm of this space is $\Vert f\Vert _{m,p} = \bigg (\displaystyle \sum \nolimits _{0 \le |\alpha | \le m} \Vert D^{\alpha }f\Vert _p^p\bigg )^{1/p}$, with *m* a positive integer and $1\le p < \infty$. A detailed presentation of Sobolev spaces can be found in [1].
- $C^m({\mathbb {T}}^2; X)$ is the (vector) space of all *X*-valued functions *f* which are continuous on ${\mathbb {T}}^2$ with continuous partial derivatives $D^{\alpha }f$ of orders $|\alpha | \le m$, for $m \ge 0$. $C^{\infty }({\mathbb {T}}^2; X)$ is regarded as the intersection of all spaces $C^{m}({\mathbb {T}}^2; X)$. Note that on the torus all continuous functions are bounded.
- $C([0, \infty ); X)$ is the space of continuous functions from $[0, \infty )$ to *X*, equipped with the uniform convergence norm over compact subintervals of $[0, \infty )$.
- $L^p(0, T; X)$ is the space of measurable functions from [0, *T*] to *X* such that the norm
  $$\begin{aligned} \Vert f\Vert _{L^p(0, T; X)}=\bigg (\displaystyle \int \limits _0^T \Vert f(t)\Vert _X^pdt\bigg )^{1/p} \end{aligned}$$
  is finite.
- $D([0, \infty ); X)$ is the space of càdlàg functions, that is functions $f:[0, \infty ) \rightarrow X$ which are right-continuous and have limits to the left, endowed with the Skorokhod topology. This topology is a natural choice in this case because its underlying metric transforms $D([0, \infty ); X)$ into a complete separable metric space. For further details see [23] Chapter 3, Section 5, pp. 117–118.
- Given $a: {\mathbb {T}}^{2}\rightarrow {\mathbb {R}}^2$, we define the differential operator  by  for any map $b: {\mathbb {T}}^{2}\rightarrow {\mathbb {R}}$ such that the scalar product $a\cdot \nabla b$ makes sense. In line with this, we use the notation  Denote the dual of  by  that is .
- For any vector $u \in {\mathbb {R}}^2$ we denote the gradient of *u* by $\nabla u = (\partial _1u, \partial _2 u)$ and the corresponding orthogonal by $\nabla ^{\perp }u = (\partial _2 u, -\partial _1 u )$.

### Remark 2

If $div \ \xi _{i}=\nabla \cdot \xi _{i}=0$, then the dual of the operator  is .

*Assumptions on the vector fields* $(\xi _i)_i$. The vector fields $\xi _i:{\mathbb {T}}^2 \rightarrow {\mathbb {R}} ^2$ are chosen to be time-independent and divergence-free and for numerical purposes they need to be specified from the underlying physics. For the analytical aspects, we assume that

4 $$\begin{aligned} \displaystyle \sum _{i=1}^{\infty } \Vert \xi _i\Vert _{k+1,\infty }^2 < \infty . \end{aligned}$$

Condition (4) ensures that for any $f \in {\mathcal {W}}^{2,2}({\mathbb {T}}^2)$, 5a5b Provided $\omega \in L^2(0, T; W^{2,2}({\mathbb {T}}^2, {\mathbb {R}}))$, condition (5a) ensures that the infinite sum of stochastic integrals6is well defined and belongs to $L^2(0, T; L^2({\mathbb {T}}^2, {\mathbb {R}}))$. Similarly, condition (5b) ensures that the process  is well-defined and belongs to $L^2(0, T; L^2({\mathbb {T}}^2, {\mathbb {R}}))$ provided the solution of the stochastic partial differential equation (1) belongs to a suitably chosen space (see Definition 3 below). In particular, the Itô correction in (2) is well-defined. The conditions above are needed also for proving a number of required a priori estimates (see Lemma 25 in the “Appendix”).

### Definition 3

- A *strong solution* of the stochastic partial differential equation (1) is an $({\mathcal {F}}_{t})_t$-adapted process $\omega :\Omega \times [0,\infty ) \times {\mathbb {T}}^{2}\rightarrow {\mathbb {R}}$ with trajectories in the space $C([0,\infty );{\mathcal {W}}^{k,2}({\mathbb {T}}^{2}))$, such that the identity^4^7 with $\omega _{|_{t=0}}=\omega _{0}$, holds ${\mathbb {P}}$-almost surely.^5^
- A *weak/distributional solution* of Eq. (1) is an $({\mathcal {F}}_t)_t$-adapted process $\omega : \Omega \times [0,\infty ) \times {\mathbb {T}}^2 \rightarrow {\mathbb {R}}$ with trajectories in the set $C([0,\infty ); L^2({\mathbb {T}}^2,{\mathbb {R}}))$, which satisfies the Eq. (1) in the weak topology of $L^2({\mathbb {T}}^2,{\mathbb {R}})$, i.e. 8 holds ${\mathbb {P}}$-almost surely for all $\varphi \in C^{\infty }({\mathbb {T}} ^2, {\mathbb {R}})$.
- A *martingale solution* of Eq. (1) is a triple $({\check{\omega }}, ({\check{W}}^i)_i), ({\check{\Omega }}, \mathcal {{\check{F}}}, \check{{\mathbb {P}}}), (\mathcal {{\check{F}}}_t)_t$ such that $({\check{\Omega }}, \mathcal {{\check{F}}}, \check{{\mathbb {P}}})$ is a probability space, $(\mathcal {{\check{F}}}_t)_t$ is a filtration defined on this space, ${\check{\omega }}$ is a continuous $(\mathcal {{\check{F}}}_t)_t$-adapted real valued process ${\check{\omega }} : \Omega \times [0,\infty ) \times {\mathbb {T}}^2 \rightarrow {\mathbb {R}}$ with trajectories in the set $C([0, \infty ); {\mathcal {W}}^{k,2}({\mathbb {T}}^2))$, $({\check{W}}^i)_i$ are independent $(\mathcal {{\check{F}}}_t)_t$-adapted Brownian motions and the identity  with $\check{\omega } _{|_{t=0}}=\check{\omega }_{0}$, holds $\check{{\mathbb {P}}}$-almost surely.^6^
- A *classical solution* of Eq. (1) is an $({\mathcal {F}}_t)_t$-adapted process $\omega : \Omega \times [0,\infty ) \times \mathbb {T }^2 \rightarrow {\mathbb {R}}$ with trajectories of class $C([0, \infty ); C^2( {\mathbb {T}}^2;{\mathbb {R}}))$.

### Remark 4

The velocity field *u* is not uniquely identified through the equation $\omega =curl\ u$. Indeed, any two velocity fields that differ by a constant will lead to the same vorticity map $\omega$. Instead we identify *u* through the “explicit” formula $u=\nabla ^{\perp }\Delta ^{-1}\omega$, see details in Remark 21 in the “Appendix”. In particular, since *u* and $\omega$ are defined in terms of partial derivatives of other functions, they must have zero average on the torus:

$$\begin{aligned} \int \limits _{{\mathbb {T}}^{2}}u^{1}\left( x\right) dx=\int \limits _{{\mathbb {T}}^{2}}u^{2}\left( x\right) dx=\int \limits _{{\mathbb {T}}^{2}}\omega \left( x\right) dx=0. \end{aligned}$$

This is due to the fact that $\omega = curl \ u = \partial _2u^1 - \partial _1 u^2$ therefore $\displaystyle \int \nolimits _{{\mathbb {T}}^2} \omega dx = \displaystyle \int \nolimits _{{\mathbb {T}}^2}\partial _2u^1 - \partial _1 u^2 dx = 0$ as we have periodic boundary conditions. Similarly, $u=\nabla ^{\perp } \psi$ where $\psi$ is the streamfunction (see the “Appendix”) and therefore $\displaystyle \int \nolimits _{{\mathbb {T}}^2} u dx = 0$. Note that if $\omega _0$ has zero average, then $\omega _t$ will have zero average, as it is immediate that all the terms appearing in the Euler equation (either (1) or (2)) have zero average.

### Remark 5

Note that $\omega _t \in {\mathcal {W}}^{k,2}({\mathbb {T}}^2)$ implies $u_t \in {\mathcal {W}}^{k+1,2}({\mathbb {T}}^2)$ (see the “Appendix” for details). By standard Sobolev embedding theorems, ${\mathcal {W}}^{k+1,2}({\mathbb {T}}^2) \hookrightarrow {\mathcal {W}}^{k,2}({\mathbb {T}}^2) \hookrightarrow L^\infty ({\mathbb {T}}^2)$ for $k\ge 2$, hence the terms  in (7), and  in (8), are well defined. However, to ensure that a *classical* solution (Definition 3.d) exists, we require $u\in C^2({{\mathbb {T}}})$ and therefore we need $k\ge 4$.

### Remark 6

Naturally, if $\omega _t$ is a strong solution in the sense of Definition 3, then it is also a weak/distributional solution. In this sense, our result enhances the solution properties presented in [12] at the expense of stronger assumptions on the initial condition of the stochastic partial differential equation, but without the need to impose the additional constraint (3). Note also that if $\omega _t$ is a weak/distributional solution with paths in $L^2(0,T; {\mathcal {W}}^{k,2}({\mathbb {T}}^2))$ then, by integration by parts, the equation has a strong solution.

## Main results

We restate the existence and uniqueness result, this time with complete details:

### Theorem 7

If $\omega _0 \in {\mathcal {W}}^{k,2}({\mathbb {T}}^2)$, then the two-dimensional stochastic Euler vorticity Eq. (1)admits a unique global $({\mathcal {F}}_t)_t$-adapted strong solution $\omega =\{\omega _t,t\in [0,\infty )\}$ with values in the space $C\left( [ 0,\infty );{\mathcal {W}}^{k,2}({\mathbb {T}}^2) \right)$. In particular, if $k\ge 4$ the solution is classical.

The proof of Theorem 7 is contained in Sects. 3.1 and 3.2 . We state next a result that shows the continuity with respect to initial conditions:

### Theorem 8

Let $\omega$, ${\tilde{\omega }}$ be two strong solutions of Eq. (1). Define *A* as the process $A_t:=\displaystyle \int \nolimits _0^t(\Vert \omega _s\Vert _{k,2}+1)ds$, for any $t\ge 0$. Then there exists a positive constant *C* independent of the two solutions, such that

9 $$\begin{aligned} {\mathbb {E}} [e^{-CA_t}||\omega _t-{\tilde{\omega }}_t||_{k-1,2}^2]\le ||\omega _0-{\tilde{\omega }}_0||_{k-1,2}^2. \end{aligned}$$

The proof of Theorem 8 is incorporated in Sect. 4.3.

### Remark 9

Insofar, Theorems 7 and 8 are less general than, for example, the corresponding results in [12], since the initial condition of Eq. (1) is assumed to be in $W^{k,2}({\mathbb {T}}^2)$. The relaxation to initial conditions in $L^{\infty }({\mathbb {T}}^2)$ is done in Sect. 7 where the well-posedness is achieved without the additional constraint (3). However, the importance of these results lies in their application to modelling and to the numerical analysis of Eq. (1). In particular, Theorem 7 states that the initial smoothness of the solution *is carried over for all times*. From a modelling perspective, this is quite important: should the vorticity of a fluid be modelled by Eq. (1), it is essential to have it uniquely defined everywhere. This is not the case if vorticity is only known to be in $L^\infty ({\mathbb {T}}^2)$. As an immediate consequence of a Sobolev embedding theorem, this is achieved, for example, if $\omega \in W^{2,2}({\mathbb {T}}^2)$. Separately, if Eq. (1) is used to model the evolution of the state in a data assimilation problem (see, e.g. [17] for further details) and the observable is, say, the fluid velocity at a given set of points, then that observable should be well defined *at those chosen points*. Again, just by assuming $\omega \in L^\infty ({\mathbb {T}})$ does not ensure this. Finally, Theorems 7 and 8 are also important when one is interested in the numerical approximation of Eq. (1). For example, when a finite element numerical approximation is used, the smoothness of the solution influences the choice of the basis and governs the rate of convergence of the numerical approximation (higher rates of convergence require higher smootheness), see [11]. Moreover, Theorem 8 is useful to transfer the convergence from local to global numerical approximations, in the required Sobolev norm.

### Remark 10

At the expense of additional technical arguments, Theorems 7 and 8 can be extended to cover solutions in $W^{k,p}({\mathbb {T}}^2)$ with $p\ge 2$. More precisely, the existence of solutions in $W^{k,p}({\mathbb {T}}^2)$ of the sequence of linearised truncated equations in Sect. 4.2 can be obtained by an extension (albeit non-trivial) of the results in [32] from deterministic to random coefficients. In addition, the results in Lemmas 24 and 25 would need to be extended to cover the necessary a priori bounds in $W^{k,p}({\mathbb {T}}^2)$.^7^

### Pathwise uniqueness of the solution of the Euler equation

The uniqueness of the solution of Eq. (1) is an immediate corollary of inequality (9) with $k=0$. However, the proof of (9) requires the existence of an approximating sequence which is constructed as part of the existence results. We present below a direct proof which avoids this, given the fact that pathwise uniqueness is required for the proof of existence of a strong (probabilistic) solution.

Suppose that Eq. (1) admits two global $({\mathcal {F}}_t)_t$-adapted solutions $\omega _1$ and $\omega _2$ with values in the space $C\left( [ 0,\infty );{\mathcal {W}}^{k,2}({\mathbb {T}}^2) \right)$ and let ${\bar{\omega }}:=\omega ^1-\omega ^2$. Consider the corresponding velocities $u^1$ and $u^2$ such that $curl \ u^1=\omega ^1$, $curl \ u^2=\omega ^2$ and ${\bar{u}}:=u^1-u^2$. Since both $\omega ^1$ and $\omega ^2$ satisfy (2), their difference satisfiesBy an application of the Itô formula one obtainsNote that the first and the last terms in the above identity are null (see Lemma 25)^8^ and thatThis is true since by the Sobolev embedding theorem (see [1] Theorem 4.12 case A) one has $\Vert \nabla \omega _{t}^1\Vert _{4} \le C \Vert \omega _t^1\Vert _{k,2}$ and using also the Biot–Savart law one has $\Vert {\bar{u}}_t\Vert _{4} \le C\Vert {\bar{u}}_t\Vert _{1,2} \le C \Vert {\bar{\omega }}_t\Vert _{2}$. Finally, observe that since $div \ u_t^2=0$. It follows that10Since we only have a priori bounds for the expected value of the process $t\rightarrow \Vert \omega _t^1\Vert _{k,2}$ and not for its pathwise values, the uniqueness cannot be deduced through a Gronwall type argument. Instead, we proceed as follows: let *A* be the process defined as $A_t:=\displaystyle \int \nolimits _0^tC\Vert \omega _s^1\Vert _{k,2}ds$, for any $t\ge 0$. This is an increasing process that stays finite ${\mathbb {P}}$-almost surely for all $t\ge 0$ as the paths of $\omega ^1$ are in $C\left( [ 0,\infty );{\mathcal {W}}^{k,2}({\mathbb {T}}^2) \right)$. By the product rule,

$$\begin{aligned} d\big (e^{-A_t}\Vert {\bar{\omega }}_t\Vert _{2}^2\big )=e^{-A_t}(d\Vert {\bar{\omega }}_t\Vert _{2}^2-C\Vert {\bar{\omega }}_t\Vert _{2}^2\Vert \omega _t^1\Vert _{k,2}dt) \le 0. \end{aligned}$$

This leads to

$$\begin{aligned} \begin{aligned} e^{-A_t}\Vert {\bar{\omega }}_t\Vert _{2}^2&=0. \end{aligned} \end{aligned}$$

We conclude that $e^{-A_t}\Vert {\bar{\omega }}_t\Vert _{2}^2=0$, and since $e^{-A_t}$ cannot be null due to the (pathwise) finiteness of $A_t$, we deduce that $\Vert {\bar{\omega }}_t\Vert _{2}^2=0$ almost surely, which gives the claim.

The above argument uses the fact that the terms  and  are well defined. In other words, even though we only wish to control the $L^2$-norm of the vorticity, we have to resort to higher order derivatives. This is permitted as we assumed that $\omega \in {\mathcal {W}}^{k,2}({\mathbb {T}}^2)$ for $k\ge 2$. By applying a similar argument, to control the ${\mathcal {W}}^{k,2}({\mathbb {T}}^2)$-norm of the vorticity we would need to control terms of the form  and . This is no longer allowed because we do not have enough smoothness in the system. To overcome this difficulty we will make use of a smooth approximating sequence for the vorticity equation, see Sect. 4.3.

#### Remark 11

The above uniqueness result is somewhat stronger than the uniqueness deduced from inequality (9). It shows that a solution of (1) will be unique in the larger space $L^{2}({\mathbb {T}}^2)$ rather than in the space ${\mathcal {W}}^{k,2}({\mathbb {T}}^2)$. Nevertheless, inequality (9) shows the Lipschitz property of the solution with respect to the initial condition in the weaker ${\mathcal {W}}^{k-1,2}({\mathbb {T}}^2)$-norm. It is not possible to show the same result in the stronger ${\mathcal {W}}^{k,2}({\mathbb {T}}^2)$-norm.

#### Remark 12

We note that, in contrast to the deterministic version of the Euler equation, the minimal *k* that ensures the existence of a strong solution is $k=2$. This is because of the occurrence of the term  in the Itô version of Eq. (1). Moreover, if we insist on the Stratonovich representation of Eq. (1), then we need to use the evolution equation of  to deduce the covariation between  and $W^i$ required for the rigorous definition of the Stratonovich integral. This, in turn, requires $k\ge 3$ as the term  appears in this evolution equation.

Nonetheless, the methodology from this paper can be used to cover initial conditions $\omega _0 \in {\mathcal {W}}^{k,2}({\mathbb {T}}^2)$ with $k<2$. In this case we have to content ourselves with weak/distributional solutions. Whilst this is not the subject of this paper, such a solution can be shown to exist as long as the product $\omega {\mathcal {L}}_u\varphi$ makes sense in a suitably chosen sense. We need to interpret the nonlinear term in a weak form as a generalised function and replace it with $\displaystyle \int \nolimits _0^t \langle \omega _s, u_s \cdot \nabla \varphi \rangle ds$. Then the same methodology can be applied as long as we can control $u\omega$ in a suitably chosen norm. In Sect. 7 we do this for the case $\omega _0 \in L^\infty ({\mathbb {T}}^2)$(which is the so-called Yudovich setting).

### Existence of the solution of the Euler equation

The existence of the solution of Eq. (1) is proved by first showing that a truncated version of it has a solution, and then removing the truncation. In particular we will truncate the non-linear term in (1) by using a smooth function $f_R$ equal to 1 on [0, *R*], equal to 0 on $[R+1, \infty )$, and decreasing on $[R, R+1]$, for arbitrary $R>0$. We then have the following:

#### Proposition 13

If $\omega _0 \in {\mathcal {W}}^{k,2}({\mathbb {T}}^2)$, then the following equation11admits a unique global $({\mathcal {F}}_t)_t$-adapted solution $\omega ^R=\{\omega ^R_t,t\in [0,\infty )\}$ with values in the space $C\left( [ 0,\infty );{\mathcal {W}}^{k,2}({\mathbb {T}}^2) \right)$. In particular, if $k\ge 4$, then the solution is classical.

#### Remark 14

The truncation in terms of the norm $\Vert \omega _t^{R}\Vert _{k-1,2}$ and not $\Vert \omega _t^{R}\Vert _{k,2}$ is not incidental as it suffices to control the norm $\Vert u_t^{R}\Vert _{k,2}$ (see Proposition 22).

We prove Proposition 13 in Sect. 4. For now let us proceed with the proof of global existence for the solution of the Euler equation (1).

#### Proposition 15

The solution of the stochastic 2D Euler equation (1) is global.

#### Proof

Define $\tau _R:=\inf _{t\ge 0}\{\Vert \omega _t^{R}\Vert _{k-1,2}\ge R\}$. Observe that on $[0,\tau _R]$, $f_R(\Vert \omega _t^{R}\Vert _{k-1,2})=1$, and therefore, on $[0,\tau _R]$ the solution of the truncated equation (11) is in fact a solution of (1) with all required properties. It therefore makes sense to define the process $\omega =\{\omega _t,t\in [0,\infty )\}$ $\omega _t=\omega _t^R$ for $t\in [0,\tau _R]$. This definition is consistent as, following the uniqueness property of the solution of the truncated equation (see Sect. 4.1), $\omega _t^{R} =\omega _t^{R'}$ for $t\in [0,\tau _{\min (R,R')}]$. The process $\omega$ defined this way is a solution of the Euler equation (1) on the interval $[0,\displaystyle \sup \nolimits _R\tau _R)$. To obtain a global solution we need to prove that $\displaystyle \sup \nolimits _{R>0}\tau _R = \infty$ ${\mathbb {P}}$-almost surely. Let ${\mathscr {A}}:=\{ w \in \Omega | \displaystyle \sup \nolimits _{R>0}\tau _R(w) < \infty \}$. Then

$$\begin{aligned} {\mathscr {A}} = \displaystyle \bigcup _{N>0} \displaystyle \{\sup _R\tau _R \le N\}=\displaystyle \bigcup _N \bigcap _R \displaystyle \{\tau _R \le N\}\end{aligned}$$

and

$$\begin{aligned} {\mathbb {P}}\left( \tau _R\le N\right) = {\mathbb {P}} \left( \displaystyle \sup _{t\in [0,N]}\Vert \omega _t^{R}\Vert _{k-1,2} > R\right) . \end{aligned}$$

In order to finish the proof of global existence we use Lemma 24 and the fact that

$$\begin{aligned} {\mathbb {P}}\left( \Vert \omega _t^R\Vert _{k-1,2} > R\right) \le \frac{{\mathbb {E}}\left[ \ln \left( \Vert \omega _t^R\Vert _{k-1,2}^2 + e \right) \right] }{R^2 + e} \le \frac{{\mathcal {C}}(\omega _0,T)}{R^2 + e} \xrightarrow [R \rightarrow \infty ]{}0. \end{aligned}$$

It follows that

$$\begin{aligned} {\mathbb {P}}\left( \bigcap _R \displaystyle \{\tau _R\le N\}\right) =\lim _{R\rightarrow \infty }{\mathbb {P}}\left( \tau _R \le N\right) =0. \end{aligned}$$

and therefore ${\mathbb {P}}({\mathscr {A}})=0$. This concludes the global existence for the solution of the Eq. (1). $\square$

## Analysis of the truncated equation

### Uniqueness of solution for the truncated equation

We use a similar strategy as the one used to prove the uniqueness of the solution of the (un-truncated) Euler Eq. (1). Suppose that Eq. (11) admits two global $({\mathcal {F}}_t)_t$-adapted solutions $\omega ^{1,R}$ and $\omega ^{2,R}$ with values in the space $C\left( [ 0,\infty );{\mathcal {W}}^{k,2}({\mathbb {T}}^2) \right)$. We prove that $\omega ^{1,R}$ and $\omega ^{2,R}$ must coincide. In the following, we will formally drop the dependence on *R* of the two solutions. As above, let ${\bar{\omega }}:=\omega ^1-\omega ^2$ and consider the corresponding velocities $u^1$ and $u^2$ such that $curl \ u^1=\omega ^1$, $curl \ u^2=\omega ^2$ and ${\bar{u}}:=u^1-u^2$. Since both $\omega ^1$ and $\omega ^2$ satisfy (11), their difference satisfieswhere $K_R(\omega )=f_R(\Vert \omega \Vert _{k-1,2})$. By an application of the Itô formula and after eliminating the null terms (see Lemma 25, Remark 28, and use the fact that $u_t^2$ is divergence-free), one obtainsOne can show that (see [18] for a proof) there exists a constant $C=C(R)$ such that

$$\begin{aligned} \Vert K_R(\omega _t^1)u^1_t-K_R(\omega _t^2){u^2_t}\Vert _4\le C\Vert {{\bar{\omega }}}_t\Vert _{k-1,2} \end{aligned}$$

and to finally deduce thatIt follows that (note that the stochastic term is null)

$$\begin{aligned} \begin{aligned} d\Vert {\bar{\omega }}_t\Vert _2^2&\le C\Vert \omega _t^1\Vert _{k,2}\Vert {\bar{\omega }}_t\Vert _{k,2}^2dt. \end{aligned} \end{aligned}$$

Similar arguments are used to control $\Vert \partial ^\alpha {\bar{\omega }}_t\Vert _2^2$ where $\alpha$ is a multi-index with $|\alpha |\le k-1$ and to deduce that there exists a constant *C* such thatwhere we use the control (see Lemma 25)Some care is required for the case when $|\alpha |= k-1$ as  is no longer well-defined. In this case, by using the weak form of the Eq. (11) to rewrite  as  we can proceed as above by using thatThe above is true for functions in ${\mathcal {W}}^{k+1,2}({\mathbb {T}}^2)$ and, by the continuity of both sides in the above inequality, it is also true for functions in the larger space ${\mathcal {W}}^{k,2}({\mathbb {T}}^2)$, since ${\mathcal {W}}^{k+1,2}({\mathbb {T}}^2)$ is dense in ${\mathcal {W}}^{k,2}({\mathbb {T}}^2)$. Using the above one can deduce that there exists a constant $C=C(R)$ such that

$$\begin{aligned} {\mathbb {E}} [e^{CA_t}||\omega _t^1-\omega _t^2||_{k-1,2}^2]\le 0, \end{aligned}$$

where *A* is the process $A_t:=\displaystyle \int _0^t(\Vert \omega _s^1\Vert _{k,2}+1)ds$ for any $t\ge 0$, hence the uniqueness of the solution of the truncated equation holds.

### Existence of solution for the truncated equation

The strategy of proving that the truncated Eq. (11) has a solution is to construct an approximating sequence of processes that will converge in distribution to a solution of (11). This justifies the existence of a weak solution. Together with the pathwise uniqueness of the solution of this equation, we then deduce that strong uniqueness holds.

Recall that $\omega _0 \in {\mathcal {W}}^{k,2}({\mathbb {T}}^2)$. Let $(\omega _0^{n})_n \in C^{\infty }({\mathbb {T}}^2)$ be a sequence such that $\omega _0^{n} \xrightarrow {n\rightarrow \infty }\omega _0$ in ${\mathcal {W}}^{k,2}({\mathbb {T}}^2)$. For any $t\ge 0$ we construct the sequence $(\omega _t^{\nu _n,R,n})_{n\ge 0}$ with $\omega _t^{\nu _0,R,0} := \omega _0^0$ and for $n \ge 1$, $\omega _0^{\nu _n,R,n} := \omega _0^n$,12where $\nu _n = \frac{1}{n}$ is the viscous parameter ($n>0$) and $u_t^{\nu _{n-1},R,n-1}=curl^{-1}(\omega _t^{\nu _{n-1},R,n-1})$.^9^ Also $K_R(\omega _t^{\nu _n,R,n}):=f_R(\Vert \omega _t^{\nu _n,R,n}\Vert _{k-1,2})$. The corresponding Itô form of Eq. (12) is^10^13where $P_t^{n-1,n}(\omega _t^{\nu _n,R,n})$ is defined as14

#### Theorem 16

If $\omega _0^{\nu _n,R,n} \in C^{\infty }({\mathbb {T}}^2)$ is a function with null spatial mean, then the two-dimensional stochastic vorticity equation (13) admits a unique global $({\mathcal {F}}_t)_t$-adapted solution $\omega ^{\nu _n,R,n}=\{\omega _t^{\nu _n,R,n},t\in [0,\infty )\}$ in the space $C\big ([ 0,\infty );C^\infty ({\mathbb {T}}^2) \big )$.

The proof of this theorem is provided in Sect. 5.

#### Proposition 17

The laws of the family of solutions $(\omega ^{\nu _n,R,n})_{\nu _n\in [0,1]}$ are relatively compact in the space of probability measures over $D([0,T],L^{2}({\mathbb {T}}^2))$ for any $T\ge 0$.

The proof of Proposition 17 is left for Sect. 6.

#### Proof of existence of the solution of Eq. (11)

Using a diagonal subsequence argument we can deduce from Proposition 17 and the fact that $\displaystyle \lim \nolimits _{n\rightarrow \infty } \omega _0^{\nu _n,R,n} =\omega _0$ the existence of a subsequence $(\omega ^{\nu _{n_j}})_{j}$ with $\displaystyle \lim \nolimits _{j\rightarrow \infty }\nu _{n_j}=0$, which is convergent in distribution over $D([0,\infty ),L^2({\mathbb {T}}^2))$. We show that the limit of the corresponding distributions is the distribution of a stochastic process that solves (11). This justifies the existence of a weak (probabilistic) solution. By using the Skorokhod representation theorem (see [9] Section 6, pp. 70), there exists a space $(\tilde{\Omega }, \tilde{{\mathcal {F}}}, \tilde{{\mathbb {P}}})$ and a sequence of processes $(\tilde{\omega }^{\nu _n,R,n}, {\tilde{u}}^{\nu _n,R,n}, ({\widetilde{W}}^{i,n})_i, n=1, \infty )$ which has the same distribution as that of the original converging subsequence and which converges (when $n \rightarrow \infty$) almost surely to a triplet $(\tilde{\omega }^R, {\tilde{u}}^R, ({\widetilde{W}}^i)_i )$ in $D([0,T],L^2({\mathbb {T}}^2)\times {\mathcal {W}}^{1,2}({\mathbb {T}}^2)\times {\mathbb {R}}^{{\mathbb {N}}})$. Note that $\omega ^{\nu _n,R,n}$ and $\tilde{\omega }^{\nu _n,R,n}$ have the same distribution, so that for any test function $\varphi \in C^{\infty }({\mathbb {T}}^2)$ we have15Note that there exists a constant $C=C(T)$ such that

16 $$\begin{aligned} \displaystyle \sup _{n\ge 1}\tilde{{\mathbb {E}}}\bigg [\displaystyle \sup _{s\in [0,T]}\Vert \tilde{\omega }^{\nu _n,R,n}_s\Vert _{k,2}^4\bigg ] \le C, \end{aligned}$$

where $\tilde{{\mathbb {E}}}$ is the expectation with respect to $\tilde{{\mathbb {P}}}$. We prove this in Lemma 25 for the original sequence, but since $\tilde{\omega }^{\nu _n,R,n}$ satisfies the same SPDE, the same a priori estimates hold for $\tilde{\omega }^{\nu _n,R,n}$. Since the space of continuous functions is a subspace of the space of càdlàg functions and the Skorokhod topology restricted to the space of continuous functions coincides with the uniform topology, it follows that the sequence $(\tilde{\omega }^{\nu _n,R,n}, {\tilde{u}}^{\nu _n,R,n}, ({\widetilde{W}}^{i,n})_i, n=1, \infty )$ converges (when $n \rightarrow \infty$) $\tilde{{\mathbb {P}}}$-almost surely to $(\tilde{\omega }^R, {\tilde{u}}^R, ({\widetilde{W}}^i)_i )$ also in the uniform norm. It also holds that

$$\begin{aligned} \lim _{n\rightarrow \infty }\tilde{{\mathbb {E}}}\left[ \int \limits _0^t || \tilde{\omega }_s^{\nu _n,R,n}-\tilde{\omega }_s^R||^2ds\right] =0 \end{aligned}$$

and since17also the limit of the right hand side of (17) converges to 0 (we use here the control  assumed in (5b)).

The convergence of the stochastic integrals in (15) is obtained by means of Theorem 32. To obtain this we use a countable dense set $(\varphi ^j)_j \in {\mathcal {W}}^{2,2}({\mathbb {T}}^2)$ and show the convergence of (15) for this particular sequence. The convergence of (15) for an arbitrary $\varphi \in {\mathcal {W}}^{2,2}({\mathbb {T}}^2)$ follows via a density argument. Let us identify precisely the sequence of processes to which we apply Theorem 32. Letwhere ${\tilde{W}}^{i,j,n}={\tilde{W}}^{i,n}$ for all $i,j,n\in {\mathbb {N}}$. Note that

$$\begin{aligned} X^n \in D\left( [0,T], L^2({\mathbb {T}}^2)\times {\mathcal {W}}^{1,2}({\mathbb {T}}^2)\times {\mathbb {R}}^{{\mathbb {N}}^2}\right) . \end{aligned}$$

From the above, the sequence $(X^n,W^n)_n$ converges in distribution to (*X*, *W*) whereand ${\tilde{W}}^{i,j}={\tilde{W}}^{i}$ for all $i,j \in {\mathbb {N}}$. Thenand, by Theorem 32,

$$\begin{aligned} \left( X^n, W^n, \displaystyle \int \limits _0^t X_s^ndW_s^n \right) _n \end{aligned}$$

converges in distribution to

$$\begin{aligned} \left( X, W, \int \limits _{0}^{t} X_sdW_s \right) , \end{aligned}$$

whereas required. By a similar application of the Skorokhod representation theorem, we can also assume that, on $(\tilde{\Omega }, \tilde{{\mathcal {F}}}, \tilde{{\mathbb {P}}})$, the above convergence holds $\tilde{{\mathbb {P}}}$-almost surely (as well as in $L^2(\tilde{{\mathbb {P}}})$).

Let us prove the convergence of the remaining terms in (15):

- $\tilde{\omega }^{\nu _n,R,n}$ converges $\tilde{{\mathbb {P}}}$-almost surely to $\tilde{\omega }^R$ in $D([0,\infty ), L^2({\mathbb {T}}^2))$. Since $\varphi$ is bounded, it follows that $\langle \tilde{\omega }_t^{\nu _n,R,n}, \varphi \rangle \xrightarrow [n \rightarrow \infty ]{} \langle {\tilde{\omega }}_t^R, \varphi \rangle$ and $\langle \tilde{\omega }_0^{\nu _n,R,n}, \varphi \rangle \xrightarrow [n \rightarrow \infty ]{} \langle {\tilde{\omega }}_0^R, \varphi \rangle$ $\tilde{{\mathbb {P}}}$-almost surely (as well as in $L^2(\tilde{{\mathbb {P}}})$), for any $\varphi \in C^{\infty }({\mathbb {T}}^2)$.
- The second term on the right hand side of (15) converges to 0 when $n \rightarrow \infty$ because the integral is uniformly bounded in $L^2(\tilde{{\mathbb {P}}})$ (again, because of (16)) and $\nu _n \rightarrow 0$ when $n \rightarrow \infty$.
- Using the fact that ${\tilde{u}}^{\nu _{n-1},R,n-1}$ is the convolution between ${\tilde{\omega }}^{\nu _{n-1},R,n-1}$ and the Biot–Savart kernel, we obtain that ${\tilde{u}}^{\nu _n,R,n}$ converges to ${\tilde{u}}^R$ $\tilde{{\mathbb {P}}}$-almost surely (as well as in $L^2(\tilde{{\mathbb {P}}})$).
- In order to show the convergence of the nonlinear term, one can write
  $$\begin{aligned} \begin{aligned}&\displaystyle \int \limits _{0}^{t}|\langle K_R(\tilde{\omega }_s^{\nu _{n-1},R,n-1}) ({\tilde{u}}_s^{\nu _{n-1},R,n-1} \cdot \nabla {\tilde{\omega }}_s^{\nu _n,R,n})- K_R(\tilde{\omega }_s^{R})({\tilde{u}}_s^R \cdot \nabla \tilde{\omega }_s^R),\varphi \rangle |ds \\&\quad = \displaystyle \int \limits _{0}^{t}K_R(\tilde{\omega }_s^{R})|\langle ({\tilde{u}}_s^{\nu _{n-1},R,n-1} - {\tilde{u}}_s^R) \cdot \nabla {\tilde{\omega }}_s^{\nu _n,R,n}, \varphi \rangle |ds \\&\qquad +\, \displaystyle \int \limits _{0}^{t}K_R(\tilde{\omega }_s^{R})|\langle {\tilde{u}}_s^R \cdot (\nabla \tilde{\omega }_s^{\nu _n,R,n} - \nabla \tilde{\omega }_s^R),\varphi \rangle | ds \\&\qquad +\, |K_R(\tilde{\omega }_s^{\nu _{n-1},R,n-1})-K_R(\tilde{\omega }_s^{R})||\langle {\tilde{u}}_s^{\nu _{n-1},R,n-1} \cdot \nabla {\tilde{\omega }}_s^{\nu _n,R,n}, \varphi \rangle |. \end{aligned} \end{aligned}$$
  For the first term we have
  $$\begin{aligned}&{\mathbb {E}}\left[ \displaystyle \int \limits _0^t K_R(\tilde{\omega }_s^R)|\langle ({\tilde{u}}_s^{\nu _{n-1},R,n} - {\tilde{u}}_s^R) \cdot \nabla \tilde{\omega }_s^{\nu _{n},R,n}, \varphi \rangle |ds \right] \\&\quad \le C(\Vert \varphi \Vert _{\infty }){\mathbb {E}}\left[ \displaystyle \sup _{s\in [0,t]}\Vert {\tilde{u}}_s^{\nu _{n-1},R,n-1} - {\tilde{u}}_s^R\Vert _2\displaystyle \int \limits _0^t\Vert \nabla \tilde{\omega }_s^{\nu _n,R,n}\Vert _2 ds\right] \\&\quad \le C(\Vert \varphi \Vert _{2,2})\sqrt{{\mathbb {E}}\left[ \displaystyle \sup _{s\in [0,t]}\Vert {\tilde{u}}_s^{\nu _{n-1},R,n-1} - {\tilde{u}}_s^R\Vert _2^2\right] {\mathbb {E}}\left[ \displaystyle \int \limits _0^t\Vert \nabla \tilde{\omega }_s^{\nu _n,R,n}\Vert _2^2 ds \right] } \\&\quad \le C(t, \Vert \varphi \Vert _{2,2})\sqrt{{\mathbb {E}}\left[ \displaystyle \sup _{s\in [0,t]}\Vert {\tilde{u}}_s^{\nu _{n-1},R,n-1} - {\tilde{u}}_s^R\Vert _2^2\right] {\mathbb {E}}\left[ \displaystyle \sup _{s\in [0,t]}\Vert \tilde{\omega }_s^{\nu _n,R,n}\Vert _{1,2}^2 \right] } \\&\quad \le C(t, \Vert \varphi \Vert _{2,2}){\mathbb {E}}\left[ \displaystyle \sup _{s\in [0,t]}\Vert {\tilde{u}}_s^{\nu _{n-1},R,n-1} - {\tilde{u}}_s^R\Vert _2^2\right] ^{1/2} \end{aligned}$$
  The term on the right hand side converges to 0 in $L^2(\tilde{{\mathbb {P}}})$ and all other terms are controlled uniformly in *n*. For the second term we have
  $$\begin{aligned} \begin{aligned}&{\mathbb {E}}\left[ \displaystyle \int \limits _0^t K_R(\tilde{\omega }_s^R)|\langle {\tilde{u}} _s^R \cdot \nabla (\tilde{\omega }_s^{\nu _{n-1},R,n-1}-\tilde{\omega }_s^R), \varphi \rangle |ds\right] \\&\quad = {\mathbb {E}}\left[ \displaystyle \int \limits _0^t K_R(\tilde{\omega }_s^R)|\langle {\tilde{u}} _s^R \cdot (\tilde{\omega }_s^{\nu _{n-1},R,n-1}-\tilde{\omega }_s^R), \nabla \varphi \rangle |ds\right] \\&\quad \le {\mathbb {E}}\left[ \displaystyle \sup _{s\in [0,t]}\Vert \tilde{\omega }_s^{\nu _{n-1},R,n-1}-\tilde{\omega }_s^R\Vert _2\displaystyle \int \limits _0^tK_R(\tilde{\omega }_s^R)\Vert {\tilde{u}}_s^R \cdot \nabla \varphi \Vert _2ds \right] \\&\quad \le C \sqrt{{\mathbb {E}}\left[ \displaystyle \sup _{s\in [0,t]}\Vert \tilde{\omega }_s^{\nu _{n-1},R,n-1}-\tilde{\omega }_s^R\Vert _2^2\right] {\mathbb {E}}\left[ \displaystyle \int \limits _0^t K_R(\tilde{\omega }_s^R)^2\Vert {\tilde{u}}_s^R\Vert _{1,2}^2\Vert \nabla \varphi \Vert _{1,2}^2\right] } \\&\quad \le C(t,\Vert \varphi \Vert _{2,2}, R){\mathbb {E}}\left[ \displaystyle \sup _{s\in [0,t]}\Vert \tilde{\omega }_s^{\nu _{n-1},R,n-1}-\tilde{\omega }_s^R\Vert _2^2\right] ^{1/2} \ \ \xrightarrow [n\rightarrow \infty ]{}0. \end{aligned} \end{aligned}$$
  For the third term, using Hölder’s inequality and an interpolation argument, we obtain:
  $$\begin{aligned} \begin{aligned}&{\mathbb {E}}\left[ \displaystyle \int \limits _0^t|K_R(\tilde{\omega }_s^{\nu _{n-1},R,n-1})-K_R(\tilde{\omega }_s^{R})||\langle {\tilde{u}}_s^{\nu _{n-1},R,n-1} \cdot \nabla \tilde{\omega }_s^{\nu _n,R.n}, \varphi \rangle |ds\right] \\&\quad \le C(\Vert \varphi \Vert _{\infty }) \sqrt{{\mathbb {E}}\left[ \displaystyle \int \limits _0^t|K_R(\tilde{\omega }_s^{\nu _{n-1},R,n-1})-K_R(\tilde{\omega }_s^{R})|^2ds\right] {\mathbb {E}}\left[ \displaystyle \int \limits _0^t\Vert {\tilde{u}}_s^{\nu _{n-1},R,n-1} \cdot \nabla \tilde{\omega }_s^{\nu _n,R,n}\Vert _2^2ds\right] } \\&\quad \le C(t,\Vert \varphi \Vert _{2,2})\\&\qquad \times \sqrt{{\mathbb {E}}\left[ \displaystyle \sup _{s\in [0,t]}\Vert \tilde{\omega }_s^{\nu _{n-1},R,n-1}{-}\tilde{\omega }_s^{R}\Vert _2^{2/k}\displaystyle \int \limits _0^t\Vert \tilde{\omega }_s^{\nu _{n-1},R,n-1}{-}\tilde{\omega }_s^{R}\Vert _{k,2}^{2(k-1)/k}ds\right] {\mathbb {E}}\left[ \displaystyle \sup _{s\in [0,t]}\Vert \omega _s^{\nu _n,R,n}\Vert _{k,2}^2\right] } \\&\quad \le C(t,\Vert \varphi \Vert _{2,2}){\mathbb {E}}\left[ \displaystyle \sup _{s\in [0,t]}\Vert \tilde{\omega }_s^{\nu _{n-1},R,n-1}-\tilde{\omega }_s^{R}\Vert _2^2\right] ^{1/2k}{\mathbb {E}}\left[ \displaystyle \int \limits _0^t(\Vert \tilde{\omega }_s^{\nu _{n-1},R,n-1}\Vert _{k,2}^2 + \Vert \tilde{\omega }_s^{R}\Vert _{k,2}^2) ds\right] ^{(k-1)/2k} \\&\quad \le {\tilde{C}}(t,\Vert \varphi \Vert _{2,2}){\mathbb {E}}\left[ \displaystyle \sup _{s\in [0,t]}\Vert \tilde{\omega }_s^{\nu _{n-1},R,n-1}-\tilde{\omega }_s^{R}\Vert _2^2\right] ^{1/2k} \ \ \xrightarrow [n\rightarrow \infty ]{}0. \end{aligned} \end{aligned}$$
- Lastly, the integrals coming from the Itô correction term are treated in a similar fashion using that:
  $$\begin{aligned} \begin{aligned}&|\langle \xi _i \cdot \nabla (\xi _i \cdot \nabla \tilde{\omega }_s^{\nu _n,R,n})- \xi _i \cdot \nabla (\xi _i \cdot \nabla {\tilde{\omega }}_s^{R}), \varphi \rangle | \\&\quad = |\langle \xi _i \cdot \nabla \tilde{\omega }_s^{\nu _n,R,n} -\xi _i \cdot \nabla {\tilde{\omega }}_s^{R}, \xi _i \cdot \nabla \varphi \rangle | \\&\quad = |\langle {\tilde{\omega }}_s^{\nu _n,R,n} - {\tilde{\omega }}_s^{R}, \xi _i \cdot \nabla (\xi _i \cdot \nabla \varphi )\rangle | \\&\quad \le \Vert \xi _i \cdot \nabla (\xi _i \cdot \nabla \varphi )\Vert _2 \Vert \tilde{\omega }_s^{\nu _n,R,n} - \tilde{\omega }_s^R\Vert _2 \xrightarrow [n\rightarrow \infty ]{}0 \end{aligned} \end{aligned}$$
  since $\Vert \xi _i \cdot \nabla (\xi _i \cdot \nabla \varphi )\Vert _2$ is finite by condition (5b) imposed initially on $(\xi _i)_i$.

We have shown so far that there exists a weak/distributional solution in the sense of Definition 3. part b. on the space $(\tilde{\Omega }, \tilde{{\mathcal {F}}}, \tilde{{\mathbb {P}}})$. However, since $\tilde{\omega }^R$ belongs to the space ${\mathcal {W}}^{k,2}({\mathbb {T}}^2) \hookrightarrow C^{k-m}({\mathbb {T}}^2)$ the solution is also strong, again, as a solution on $(\tilde{\Omega }, \tilde{{\mathcal {F}}}, \tilde{{\mathbb {P}}})$ (and not on the original space). It follows that $(\tilde{\omega }, {\tilde{u}}, ({\widetilde{W}}^i)_i)$ is a martingale solution of the truncated Euler equation (11) in the sense of Definition 3 part c. Together with the pathwise uniqueness proved in Sect. 4.2 and using the Yamada-Watanabe theorem for the infinite-dimensional setting (see, for instance, [48]) we conclude the existence of a strong solution of the truncated Euler equation.

From the above, it follows that the limiting process is continuous in the $L^2$-norm and, by interpolation, in the ${\mathcal {W}}^{k-\varepsilon ,2}$-norm for any $\varepsilon >0$. To show continuity in the ${\mathcal {W}}^{k,2}$-norm we follow the standard argument: we observe first that the sequence is weakly continuous in this norm, so it suffices to show that the ${\mathcal {W}}^{k,2}$-norm of the process is continuous. For this we apply the Kolmogorov-Čentsov criterion. By Fatou’s lemma, we have that

$$\begin{aligned} {\mathbb {E}}[(\Vert \omega _t\Vert _{k,2}^2 - \Vert \omega _s\Vert _{k,2}^2)^4] \le \liminf _{n\rightarrow \infty } {\mathbb {E}}[(\Vert \omega _t^{\nu _n,R,n}\Vert _{k,2}^2 - \Vert \omega _s^{\nu _n,R,n}\Vert _{k,2}^2)^4] \end{aligned}$$

and the right hand side of the above inequality can be controlled by $C(t-s)^2$ using an argument similar to the one from Sect. 4.3, where *C* is a constant independent of *s*, *t* and *n*. Therefore $\omega ^R \in C([0,T], {\mathcal {W}}^{k,2}({\mathbb {T}}^2))$^11^ and the solution exists in the sense of Definition 3, part a.

Now using the embedding ${\mathcal {W}}^{k,2}({\mathbb {T}}^2) \hookrightarrow C^{k-m}({\mathbb {T}}^2)$ with $\ 2\le m \le k$ and $k\ge 4$ we conclude that the solution is classical when $k \ge 4$.

### Proof of Theorem 8

We are finally ready to show continuity with respect to initial conditions. As stated in the theorem, let $\omega$, ${\tilde{\omega }}$ be two $C\left( [ 0,\infty );{\mathcal {W}}^{k,2}({\mathbb {T}}^2) \right)$-solutions of Eq. (1) and define *A* as the process $A_t:=\displaystyle \int _0^t(\Vert \omega _s\Vert _{k,2}+1)ds$, for any $t\ge 0$. Let $\omega ^R$, ${\tilde{\omega }}^R$ be their corresponding truncated versions and also let $(\omega _t^{\nu _n,R,n})_{n\ge 0}$ and $({\tilde{\omega }}_t^{\nu _n,R,n})_{n\ge 0}$ be, respectively, the corresponding sequences constructed as in Sect. 4.2 on the same space after the application of the Skorokhod representation theorem. By Fatou’s lemma, applied twice, we deduce that

$$\begin{aligned} \begin{aligned} {\mathbb {E}} [e^{-CA_t}||\omega _t-{\tilde{\omega }}_t||_{k-1,2}^2]&\le {\mathbb {E}} \big [\displaystyle \liminf _n e^{-CA^n_t}||\omega _t^{\nu _n,R,n}-{\tilde{\omega }}_t^{\nu _n,R,n}||_{k-1,2}^2\big ] \\&\le \displaystyle \liminf _n {\mathbb {E}} [e^{-CA^n_t}||\omega _t^{\nu _n,R,n}-{\tilde{\omega }}_t^{\nu _n,R,n}||_{k-1,2}^2] \end{aligned} \end{aligned}$$

where $A^n$ is the process defined by $A_t^n:=\displaystyle \int _0^t(\Vert \omega _s^{\nu _n,R,n}\Vert _{k,2}+1)ds$, for any $t\ge 0$. Following a similar proof with that of the uniqueness of the Euler equation, one then deduces that there exists a positive constant *C* independent of the two solutions and independent of *R* and *n* such that

$$\begin{aligned} {\mathbb {E}} [e^{-CA^n_t}||\omega _t^{\nu _n,R,n}-{\tilde{\omega }}_t^{\nu _n,R,n}||_{k-1,2}^2]\le ||\omega _0-{\tilde{\omega }}_0||_{k-1,2}^2, \end{aligned}$$

which gives the result. We emphasize that we use here the fact that the processes $(\omega _t^{\nu _n,R,n})_{n\ge 0}$ and $({\tilde{\omega }}_t^{\nu _n,R,n})_{n\ge 0}$ take values in ${\mathcal {W}}^{k+2,2}({\mathbb {T}}^2)$ as an essential ingredient, a property that was not true for either the solution of the Euler equation or its truncated version.

## Existence, uniqueness, and continuity of the approximating sequence of solutions

### Existence and uniqueness of the approximating sequence

We show that the sequence $(\omega _t^{\nu _n,R,n})_n$ given by formula (13), that is,with $\omega _0^{\nu _n,R,n}=\omega _0^n$, is smooth. The equation above is a particular case of Eq. (1.1)–(1.2) in Chapter 4, Section 4.1, p. 129 in [49]. All assumptions required by Theorem 1 and Theorem 2 in [49], Chapter 4, are fulfilled. Therefore there exists a unique solution $\omega _t^{\nu _n,R,n}$ which belongs to the class $L^2(0,T; {\mathcal {W}}^{k,2}({\mathbb {T}}^2)) \cap C([0,T], {\mathcal {W}}^{k-1,2}({\mathbb {T}}^2))$ and satisfies Eq.  (13) for all $t\in [0,T]$ and for all $\omega$ in $\Omega ' \subset \Omega$ with ${\mathbb {P}}(\Omega ') = 1.$

Furthermore, since the conditions are fulfilled for all $k\in {\mathbb {N}}$, using Corollary 3 from p. 141 in [49], we obtain that $\omega _t^{\nu _n,R,n}$ is ${\mathbb {P}}$—a.s. in $C\big ([0,T], C^{\infty }({\mathbb {T}}^2)\big )$. Note that $u_t^{\nu _{n-1},R,n-1} \in C^{\infty }({\mathbb {T}}^2)$ for any $n \ge 1$, using the Biot–Savart law and an inductive argument. One has $u_t^{\nu _{n-1},R,n-1} = K \star \omega _t^{\nu _{n-1},R,n-1}$ with *K* being the Biot–Savart kernel defined in the “Appendix”. The convolution between *K* and $\omega _t^{\nu _{n-1},R,n-1}$ is commutative, so we have

$$\begin{aligned} u_t^{\nu _{n-1},R,n-1}(x) = \displaystyle \int \limits _{{\mathbb {T}}^2}K(y)\omega _t^{\nu _{n-1},R,n-1}(x-y)dy. \end{aligned}$$

Since $\omega _t^{\nu _{n-1}}$ is in $C^{\infty }({\mathbb {T}}^2)$ by Corollary 3 (at step $n-1$), and using the fact that $K\in L^1({\mathbb {T}}^2)$, we conclude that $u_t^{\nu _{n-1},R,n-1}\in C^{\infty }({\mathbb {T}}^2)$. This, together with the initial Assumption (4), ensures that all the coefficients of Eq. (13) are infinitely differentiable. The uniform boundedness is ensured by the truncation $K_R(\omega _t^{\nu _{n-1},R,n-1})$, as proven in Lemma 25 from the “Appendix”.

### Continuity of the approximating sequence

#### Proposition 18

There exists a constant $C=C(T)$ independent of *n* and *R* such that

$$\begin{aligned} {\mathbb {E}}[\Vert \omega _t^{\nu _n,R,n} - \omega _s^{\nu _n,R,n}\Vert _{L^2}^4] \le C(t-s)^2,\ \ t,s\in [0,T]. \end{aligned}$$

In particular, by the Kolmogorov-Čentsov criterion (see [37]), the processes $\omega ^{\nu _n,R,n}$ have continuous trajectories in $L^2({\mathbb {T}}^2)$.

#### Proof

Consider $s\le t$. Then18We will estimate the expected value of each of these terms. For the first term we have

$$\begin{aligned} \begin{aligned} {\mathbb {E}}\bigg [\bigg \Vert \displaystyle \int \limits _s^t\nu _n \Delta \omega _p^{\nu _n,R,n}dp \bigg \Vert _{L^2}^4\bigg ]&\le (t-s)^4{\mathbb {E}}[\displaystyle \sup _{p\in [s,t]}\Vert \Delta \omega _p^{\nu _n,R,n}\Vert _2^4] \\&\le T^{2}(t-s)^2{\mathbb {E}}[\displaystyle \sup _{p\in [0,t]}\Vert \omega _p^{\nu _n,R,n}\Vert _{k,2}^4]\\&\le C(t-s)^2. \end{aligned} \end{aligned}$$

Next we have19The penultimate inequality is true given that

$$\begin{aligned}&\Vert K_R(\omega _p^{\nu _{n-1},R,n-1})u_p^{\nu _{n-1},R,n-1} \cdot \nabla \omega _p^{\nu _n,R,n}\Vert _{L^2}^2 \le C\Vert u_p^{\nu _{n-1},R,n-1}\Vert _{\infty }^2\Vert \nabla \omega _p^{\nu _n,R,n}\Vert _2^2 \\&\quad \le C\Vert \omega _p^{\nu _n,R,n}\Vert _{k,2}^2 \end{aligned}$$

since

$$\begin{aligned} \Vert \nabla u_p^{\nu _{n-1},R,n-1}\Vert _{\infty }\le & {} C\Vert \nabla u_p^{\nu _{n-1},R,n-1}\Vert _{k,2}\le C \Vert u_p^{\nu _{n-1},R,n-1}\Vert _{k+1,2}\\\le & {} C\Vert \omega _p^{\nu _{n-1},R,n-1}\Vert _{k,2}, \end{aligned}$$

and the last term is finite in expectation due to the a priori estimates proved in Lemma 25 vi. Similarly, we can prove thatwhich, together with (19) gives a control on the second term of (18). For the last term we use the Burkholder–Davis–Gundy inequality and obtain

$$\begin{aligned} \begin{aligned} {\mathbb {E}}\bigg [\bigg \Vert \displaystyle \int \limits _s^t\displaystyle \sum _{i=1}^{\infty } \xi _i \cdot \nabla \omega _p^{\nu _n,R,n} dW_p^{i,n}\bigg \Vert _{L^2}^4\bigg ]&\le C{\mathbb {E}}\bigg [\bigg (\displaystyle \int \limits _s^t \displaystyle \sum _{i=1}^{\infty } \Vert \xi _i \cdot \nabla \omega _p^{\nu _n,R,n}\Vert _2^2 dp\bigg )^2\bigg ] \\&\le C(t-s)^2{\mathbb {E}}\big [\displaystyle \sup _p\displaystyle \sum _{i=1}^{\infty } \big \Vert \xi _i \cdot \nabla \omega _p^{\nu _n,R,n}\big \Vert _2^4\big ] \\&\le C(t-s)^2 {\mathbb {E}}\big [\displaystyle \sup _p\Vert \omega _p^{\nu _{n,R,n}}\Vert _{k,2}^4\big ] \\&\le C(t-s)^2 \end{aligned} \end{aligned}$$

due to the initial Assumption (4) and the a priori estimates (25). The conclusion now follows by a direct application of the Kolmogorov-Čentsov criterion. $\square$

## Relative compactness of the approximating sequence of solutions

In this section we prove that the approximating sequence of solutions constructed in Sect. 4.2 is relatively compact in the space $D([0,T], L^2({\mathbb {T}}^2))$.^12^

### Proof of Proposition 17

In order to prove relative compactness we use Kurtz’ criterion for relative compactness. For completeness we state the result in “Appendix”, see Theorem 29. To do so we need to show that, for every $\eta >0$ there exists a compact set $K_{\eta , t} \subset L^2({\mathbb {T}}^2)$ such that $\displaystyle \sup \nolimits _{n}{\mathbb {P}}\big (\omega _t^{\nu _n,R,n}\notin K_{\eta , t}\big ) \le \eta .$ The compact we use is

$$\begin{aligned} K_{\eta , t} :=\left\{ \omega \in {\mathcal {W}}^{k,2}({\mathbb {T}}^2) | \ \ \Vert \omega \Vert _{k,2} <\left( \frac{C}{\eta }\right) ^{\frac{1}{4}} \right\} \end{aligned}$$

where *C* is the constant appearing in the a priori estimates (25). By a Sobolev compact embedding theorem, $K_{\eta , t}$ is a compact set in $L^2({\mathbb {T}}^2)$ and

$$\begin{aligned}&\displaystyle \sup _{n}{\mathbb {P}}\big (\omega _t^{\nu _n,R,n}\notin K_{\eta ,t}\big ) = \displaystyle \sup _{n}{\mathbb {P}}\left( \Vert \omega _t^{\nu _n,R,n}\Vert _{k,2} \ge \left( \frac{C}{\eta }\right) ^{\frac{1}{4}} \right) \\&\le \displaystyle \sup _{n}\frac{\eta }{C}{\mathbb {E}}\left[ \sup _{t\in [0,T]}\Vert \omega _t^{\nu _n,R,n}\Vert _{k,2}^4 \right] \le \eta . \end{aligned}$$

To prove relative compactness, we need to justify part b) of Kurtz’ criterion, as per Theorem 29. For this we will show that there exists a family $(\gamma _{\delta }^n)_{0<\delta <1}$ of nonnegative random variables such that

$$\begin{aligned} {\mathbb {E}} \big [\Vert \omega _{t+l}^{\nu _n,R,n} - \omega _t^{\nu _n,R,n}\Vert _{2}^2 | {\mathcal {F}}_t^{n}\big ] \le {\mathbb {E}}\big [ \gamma _{\delta }^{n} | {\mathcal {F}}_t^{n} \big ] \end{aligned}$$

with $0 \le \ell \le \delta$ and $\displaystyle \lim \nolimits _{\delta \rightarrow 0} \sup \nolimits _{n} {\mathbb {E}}\big [ \gamma _{\delta }^{n} \big ] =0$ for $t \in [0, T]$. The filtration $({\mathcal {F}}_t^n)_{t}$ corresponds here to the natural filtration $({\mathcal {F}}_t^{\omega ^{\nu _n,R,n}})_t$. We will use the mild form of Eq. (13), that iswith $P_s^{n-1,n}$ as defined in (14) and $S^n(t):=e^{\nu _n\Delta t}$. One has20We will estimate each term separately. For the first term we have

$$\begin{aligned} \begin{aligned} {\mathbb {E}}\big [\Vert (S^n(t+l)-S^n(t))\omega _0^{\nu _n,R,n}\Vert _{2}^2|{\mathcal {F}}_t^n\big ]&\le \Vert (S^n(l)-1)\omega _0^{\nu _n,R,n}\Vert _{2}^2 \end{aligned} \end{aligned}$$

For the second term,

$$\begin{aligned} \begin{aligned}&{\mathbb {E}}\bigg [\bigg \Vert \displaystyle \int \limits _0^t(S^n(t+l-s)-S^n(t-s))P_s^{n-1,n}(\omega _s^{\nu _n,R,n})ds\bigg \Vert _{2}^2 \bigg | {\mathcal {F}}_t^n\bigg ] \\&\quad \le T{\mathbb {E}}\bigg [ \displaystyle \int \limits _0^T \Vert (S^n(l)-1)P_s^{n-1,n}(\omega _s^{\nu _n,R,n})\Vert _{2}^2 ds \bigg | {\mathcal {F}}_t^n\bigg ] \end{aligned} \end{aligned}$$

For the third term we have

$$\begin{aligned} \begin{aligned}&{\mathbb {E}}\bigg [\bigg \Vert \displaystyle \int \limits _t^{t+l}S^n(t+l-s)P_s^{n-1,n}(\omega _s^{\nu _n,R,n})ds\bigg \Vert _{2}^2 \bigg | {\mathcal {F}}_t^n\bigg ]\\&\quad \le {\mathbb {E}}\bigg [l^2\displaystyle \sup _{s\in [t,t+l]}\Vert S^n(t+l-s)P_s^{n-1,n}(\omega _s^{\nu _n,R,n})\Vert _{2}^2 \bigg | {\mathcal {F}}_t^n\bigg ]\\&\quad \le {\mathbb {E}}\bigg [C l^2\displaystyle \sup _{s\in [t,t+l]} \Vert P_s^{n-1,n}(\omega _s^{\nu _n,R,n})\Vert _{2}^2 \bigg | {\mathcal {F}}_t^n\bigg ]\\&\quad \le {\mathbb {E}}\bigg [Cl^2\displaystyle \sup _{s \in [0, T+1]}\Vert P_s^{n-1,n}(\omega _s^{\nu _n,R,n})\Vert _{2}^2 \bigg | {\mathcal {F}}_t^n\bigg ] \end{aligned} \end{aligned}$$

Aiming to construct the family $(\gamma _{\delta }^n)_{0<\delta <1}$ of nonnegative random variables, we still need to control the two stochastic terms. The first one is more delicate and in order to obtain a suitable control on it we will use the so-called *factorisation formula* (see e.g. [20] Section 5.3.1). More precisely, we use the fact that

$$\begin{aligned} \displaystyle \int \limits _{a_1}^{a_2}(a_2-r)^{\alpha -1}(r-a_1)^{-\alpha }dr = C({\alpha }) \end{aligned}$$

where $C(\alpha )$ is a constant which depends on $\alpha >0$ only. Using also the semigroup property $S^n(t-s) = S^n(t-r)S^n(r-s)$ for $s<r<t$, one can writewhereWe choose $\alpha \in (0, 1/2)$ such that all integrals are well-defined. Now the fourth term in (20) can be estimated as follows:and thereforeFor the fifth term in (20) we havesoWe can now defineand $\gamma _{\delta }^{n} := \displaystyle \sup \nolimits _{l \in [0, \delta ]}\gamma _l^{\nu _n}.$ From Lemma 25 we deduce that there exist two constants $c_1$ and $c_2$ such that ${\mathbb {E}}[ \displaystyle \sup _s\Vert P_s^{n-1,n}(\omega _s^{\nu _n,R,n})\Vert _{2}^2] \le c_1$ and  The integrands in the integrals above converge pointwise to 0 when $l \rightarrow 0$ due to the strong continuity of the semigroup $S^n$. At the same time, they are bounded by integrable functions, therefore the convergence is uniform in space by the dominated convergence theorem. Then the requirement

$$\begin{aligned} \displaystyle \lim _{\delta \rightarrow 0} \sup _{n} {\mathbb {E}}\big [ \gamma _{\delta }^{n} \big ] =0 \end{aligned}$$

is met. In conclusion all the conditions required by Kurtz’ criterion are fulfilled and therefore $(\omega _t^{\nu _n, R, n})_{\nu _n}$ is relatively compact. $\square$

## Recovering the solution of the Euler equation in the Yudovich setting

As an application of Theorem 7, we prove existence of the solution of the stochastic Euler equation under the relaxed assumption that $\omega _{0}\in L^{\infty }({\mathbb {T}}^{2})$. This is the so-called Yudovich setting, see e.g. [52] or [44] Section 8.2. By doing so, we duplicate the result in [12] without the need to impose Assumption (3). The result is as expected: we show existence of a weak solution $\omega _{t}\in L^{\infty }({\mathbb {T}}^{2})$ of Eq. (1 ) re-cast in its weak form (8). Note that whilst the definition of a weak solution only requires $\omega _{t}\in L^{2}({\mathbb {T}}^{2})$, we are showing here that Eq. (1) has a solution in the (smaller) space $L^{\infty }({\mathbb {T}}^{2})$.

The strategy is quite similar with that employed for proving Theorem 7. We briefly explain here the main steps without going into details. For an arbitrary $\omega _{0}\in L^{\infty }({\mathbb {T}}^{2})$, let $(\omega _{0}^{n})_{n}\in {\mathcal {W}}^{k,2}({\mathbb {T}}^{2})$ be a uniformly bounded sequence such that $\omega _{0}^{n}$ converges to $\omega _{0}$ almost surely. Consider next the sequence of strong solutions $(\omega ^{n})_{n}\in {\mathcal {W}}^{k,2}({\mathbb {T}}^{2})$ of Eq. (1), with the corresponding velocities $(u^{n})_{n}\in {\mathcal {W}}^{k+1,2}({\mathbb {T}}^{2}).$ Then $(\omega ^{n})_{n}$ is tight as a sequence with paths in $C([0,T];{\mathcal {W}}^{-2,2}({{\mathbb {T}}}^{2}))$ (see Lemma 19 below). We can therefore extract a subsequence (we re-index it if necessary) $(\omega ^{n})_{n}$ which converges in distribution to $\omega$ over the space $C([0,T];{\mathcal {W}}^{-2,2}({{\mathbb {T}}}^{2}))$. By appealing to Theorem 32 (via another reduction to a subsequence, see Sect. 4.2) we can deduce the convergence of the stochastic integrals. Consider a countable dense set $(\varphi ^j)_j \in {\mathcal {W}}^{4,2}({\mathbb {T}}^2)$. We show the convergence using this sequence, and then the convergence for an arbitrary $\varphi \in {\mathcal {W}}^{4,2}({\mathbb {T}}^2)$ follows via a density argument. Letwhere $W^{i,j,n}=W^{i,n}$ for all $i,j,n\in {\mathbb {N}}$. The sequence $(X^n,W^n)_n$ converges in distribution to (*X*, *W*), whereand $W^{i,j}=W^{i}$ for all $i,j\in {\mathbb {N}}$. Thenand, by Theorem 32,

$$\begin{aligned} \left( X^n, W^n, \displaystyle \int \limits _0^t X_s^ndW_s^n \right) _n \end{aligned}$$

converges in distribution to

$$\begin{aligned} \left( X, W, \int \limits _{0}^{t} X_sdW_s \right) \end{aligned}$$

whereNote that the stochastic integrals that appear above are interpreted as ${\mathcal {W}}^{-2,2}({\mathbb {T}}^2)$-processes. Then, using a Skorokhod representation argument similar to the one in Sect. 4.2, we deduce that the limiting process $\omega$ indeed satisfies the Itô version of Eq. (1). We do this by showing that every term in the equation satisfied by $\omega ^{n}$ converges to the corresponding term in the equation satisfied by $\omega$. The only difficult term is the nonlinear term, which we analyse in Lemma 20 below. This justifies the existence of a martingale solution of Eq. (1) in $C([0,T];{\mathcal {W}}^{-2,2}({{\mathbb {T}}}^{2}))\cap L^{\infty }(0,T;L^{\infty }({{\mathbb {T}}}^{2}))$ which together with the uniqueness of the weak solution of (1) (we use a similar argument as that for Theorem 7) provides the existence of a probabilistically strong solution.

To complete the argument we need to prove that the sequence $\left( \omega ^{n}\right) _{n}$ is tight as a sequence with paths in $C([0,T];{\mathcal {W}}^{-2,2}({{\mathbb {T}}}^{2}))$. The main ingredient in the argument is to show that, just as in the deterministic case, the vorticity $\omega ^{n}$ is propagated by inviscid flows, and therefore its $L^{p}$-norm is conserved for any $p\in (0,\infty ]$. For this we characterize the trajectories of the Lagrangian fluid particles as the solutions of the following stochastic flow

21 $$\begin{aligned} dX_{t}^{n}\left( x\right) =u_{t}^{n}\left( X_{t}^{n}\left( x\right) \right) dt+\sum _{i=1}^{\infty }\xi _{i}(X_{t}^{n}\left( x\right) )\circ dW_{t}^{i,n},~X_{0}^{n}\left( x\right) =x\text {, }~x\in {\mathbb {T}}^{2}. \end{aligned}$$

Recall that, following from Theorem 7, $(\omega ^{n})_{n}$ has paths in ${\mathcal {W}}^{k,2}({\mathbb {T}}^{2})$ with the corresponding velocities $(u^{n})_{n}$ having paths in ${\mathcal {W}}^{k+1,2}({\mathbb {T}} ^{2})$. By Theorem 4.6.5 in [40] the mapping $x\rightarrow X_{t}^{n}\left( x\right)$ is a $C^{k-1}$-diffeomorphism, for all $n\in {\mathbb {N}}$.^13^ The stochastic process $X_{t}^{n}\left( x\right)$ models the evolution of the Lagrangian particle path corresponding to a fluid parcel starting from an arbitrary value $~x\in {\mathbb {T}}^{2}$. Each Lagrangian path evolves according to a mean drift flow perturbed by a random flow which aims to model the rapid oscillations around the mean. By using the Itô-Wentzel formula, see e.g. [37], page 156, one shows that

$$\begin{aligned} d(\omega _{t}^{n}\left( X_{t}^{n}\left( x\right) \right) )=\nabla \omega _{t}^{n}\left( X_{t}^{n}\left( x\right) \right) \circ dX_{t}^{n}+d\omega _{t}^{n}\left( X_{t}^{n}\left( x\right) \right) =0. \end{aligned}$$

It follows that $\omega _{t}^{n}\left( X_{t}^{n}\left( x\right) \right) =\omega _{0}^{n}\left( x\right) \iff \omega _{t}^{n}\left( x\right) =\omega _{0}^{n}\left( X_{t}^{-n}\left( x\right) \right)$. That is, just as in the deterministic case, the vorticity is conserved along the particle trajectories. In particular, this implies that, pathwise,

22 $$\begin{aligned} \left| \left| \omega _{t}^{n}\right| \right| _{\infty }=\left| \left| \omega _{0}^{n}\right| \right| _{\infty },~~~t\ge 0. \end{aligned}$$

In other words, the vorticity remains uniformly bounded for all times and all realizations.

Next, let *h* be any measurable function such that $x\rightarrow h\left( \omega _{t}^{n}\left( x\right) \right)$ is integrable over the torus. Then

$$\begin{aligned} \int \limits _{{\mathbb {T}}^{2}}h\left( \omega _{t}^{n}\left( x\right) \right) dx= & {} \int \limits _{ {\mathbb {T}}^{2}}h\left( \omega _{0}^{n}\left( X_{t}^{-n}\left( x\right) \right) \right) dx\\= & {} \int \limits _{{\mathbb {T}}^{2}}h\left( \omega _{0}^{n}\left( x\right) \right) \det \left( J_{t}\left( x\right) \right) dx=\int \limits _{{\mathbb {T}} ^{2}}h\left( \omega _{0}^{n}\left( x\right) \right) dx \end{aligned}$$

since the determinant of the Jacobian is zero, the fluid being incompressible. In particular,

23 $$\begin{aligned} \left| \left| \omega _{t}^{n}\right| \right| _{p}=\left| \left| \omega _{0}^{n}\right| \right| _{p},~~~t\ge 0,~~~p\in (0,\infty ]. \end{aligned}$$

In addition, following the same arguments as in the deterministic case (for further details and proofs see, for example, [44], pp. 20-23), one can deduce that the vortex lines move with the solution of the stochastic Euler flow, and that Kelvin’s conservation of circulation is satisfied.^14^

### Lemma 19

The sequence $(\omega ^{n})_{n}$ as defined above is tight as a sequence with paths in $C([0,T];{\mathcal {W}}^{-2,2}({{\mathbb {T}}}^{2}))$.

### Proof

Let ${\mathcal {Z}}$ be the following space^15^

$$\begin{aligned} {\mathcal {Z}}=\left\{ f\in L^{\infty }\left( 0,T;L^{2}\left( {\mathbb {T}} ^{2}\right) \right) \cap C\left( [0,T];{\mathcal {W}}^{-2,2}\left( {\mathbb {T}}^{2}\right) \right) |\ \ \Vert f\Vert _{{\mathcal {Z}}}<\infty \right\} \end{aligned}$$

where

$$\begin{aligned} \Vert f\Vert _{{\mathcal {Z}}}^{4}:=\sup _{t\in \left[ 0,T\right] }\left\| f\left( t\right) \right\| _{L^{2}\left( {\mathbb {T}}^{2}\right) }^{4}+\int \limits _{0}^{T}\int \limits _{0}^{T}\frac{\left\| f\left( t\right) -f\left( s\right) \right\| _{{\mathcal {W}}^{-2,2}}^{4}}{\left| t-s\right| ^{2}}dtds\ \ . \end{aligned}$$

Let $B_{{\mathcal {Z}}}\left( 0,R\right) \subset {\mathcal {Z}}$ be the closed ball of radius *R* in the ${\mathcal {Z}}$-norm. Then $B_{{\mathcal {Z}}}\left( 0,R\right)$ is a compact set in $C\left( [0,T];{\mathcal {W}}^{-2,2}\left( {\mathbb {T}} ^{2}\right) \right)$. This follows from the generalized Arzela-Ascoli theorem see Lemma 1 in [50] (use the fact that $L^{2}\left( {\mathbb {T}} ^{2}\right)$ is compactly embedded in ${\mathcal {W}}^{-2,2}\left( {\mathbb {T}}^{2}\right)$) combined with Lemma 5 in [50]). The tightness then follows as

$$\begin{aligned} \lim _{R\rightarrow \infty }\sup _{n}{\mathbb {P}}\left( \omega ^{n}\in B_{{\mathcal {Z}} }\left( 0,R\right) \right) =\lim _{R\rightarrow \infty }\sup _{n}\frac{{\mathbb {E}}\left[ \Vert \omega ^{n}\Vert _{{\mathcal {Z}}}^{4}\right] }{R^{4}}=0. \end{aligned}$$

This is true as $\sup \nolimits _{n}{\mathbb {E}}[\Vert \omega ^{n}\Vert _{{\mathcal {Z}} }^{4}]<\infty$. To show the latter claim use the fact that $\sup \nolimits _{n}\Vert \omega ^{n}\Vert _{2}<\infty$ due to the existence of the stochastic flow (see above) and that there exists a constant $C=C\left( T\right)$ independent of n such that

$$\begin{aligned} {\mathbb {E}}\left[ \left\| \omega _{t}^{n}-\omega _{s}^{n}\right\| _{{\mathcal {W}}^{-2,2}}^{4} \right] \le C\left| t-s\right| ^{2}. \end{aligned}$$

$$\square $$

### Lemma 20

Let $(\omega ^{n})_{n}$ be the sequence constructed above via the Skorokhod representation theorem, and $\varphi \in {\mathcal {W}}^{1,2}({\mathbb {T}}^{2})$. Then24

### Proof

From (23), the sequence $(\omega ^{n})_{n}$ is uniformly bounded and converges to $\omega$ almost surely in $C\left( [0,T];{\mathcal {W}}^{-2,2}\left( {\mathbb {T}}^{2}\right) \right)$. Let

$$\begin{aligned} M:=\sup _{n>0}\sup _{t\in \left[ 0,T\right] }\left| \left| \omega _{t}^{n}\right| \right| _{L^{2}\left( {\mathbb {T}}^{2}\right) }<\infty . \end{aligned}$$

Also $\sup \nolimits _{t\in \left[ 0,T\right] }\left| \left| \omega _{t}\right| \right| _{L^{2}\left( {\mathbb {T}}^{2}\right) }\le M.~$We first deduce that $(\omega _{t}^{n})_{n}$ converges to $\omega _{t}$ in $L_w^{2}\left( {\mathbb {T}}^{2}\right)$ almost surely. To see this, choose an arbitrary $\varphi$ and $\varepsilon >0.$ Let $\varphi ^{\varepsilon }\in {\mathcal {W}}^{2,2}\left( {\mathbb {T}}^{2}\right)$ such that $\left| \left| \varphi -\varphi ^{\varepsilon }\right| \right| _{L^{2}\left( {\mathbb {T}}^{2}\right) }<\varepsilon /4M$ and *N* such that $\left| \left( \omega _{t}^{n},\varphi ^{\varepsilon }\right) -\left( \omega _{t},\varphi ^{\varepsilon }\right) \right| <\varepsilon /2$ for all $n\ge N$. Then, for all $n\ge N,$we have

$$\begin{aligned}&\left| \left( \omega _{t}^{n},\varphi \right) -\left( \omega _{t},\varphi \right) \right| \\&\quad =\left| \left( \omega _{t}^{n},\varphi \right) -\left( \omega _{t}^{n},\varphi ^{\varepsilon }\right) +\left( \omega _{t},\varphi ^{\epsilon } \right) -\left( \omega _{t},\varphi \right) +\left( \omega _{t}^{n},\varphi ^{\varepsilon }\right) -\left( \omega _{t},\varphi ^{\varepsilon }\right) \right| \\&\quad \le \left( \left| \left| \omega _{t}^{n}\right| \right| _{L^{2}\left( {\mathbb {T}}^{2}\right) }+\left| \left| \omega _{t}\right| \right| _{L^{2}\left( {\mathbb {T}}^{2}\right) }\right) \left| \left| \varphi -\varphi ^{\varepsilon }\right| \right| _{L^{2}\left( {\mathbb {T}}^{2}\right) }\\&\qquad +\left| \left( \omega _{t}^{n},\varphi ^{\varepsilon }\right) -\left( \omega _{t},\varphi ^{\varepsilon }\right) \right| <\varepsilon , \end{aligned}$$

hence the claim. $\square$

Next observe that, since $$u^{n}=K\star \omega ^{n}$$, where *K* is the Biot–Savart kernel defined in (25), we can deduce that $$(u^{n})_{n}$$ is uniformly bounded and converges to $$u:=K\star \omega $$ almost surely. Moreover it is uniformly continuous (see Corollary 2.18 in [12]). By Arzela–Ascoli theorem we deduce that *u* is continuous and $$ u^{n}$$ converges uniformly to *u* almost surely. More precisely we have, almost surely,

$$\begin{aligned} \sup _{t\in \left[ 0,T\right] }\left| \left| u_{t}^{n}-u_{t}\right| \right| _{L^{\infty }\left( {\mathbb {T}} ^{2}\right) }=0. \end{aligned}$$

ThenThe first term converges to 0 as $u^{n}$ converges uniformly to *u*. The second term converges to 0 as $(\omega ^{n})_n$ is uniformly bounded and converges to $\omega$ in $L_w^{2}\left( {\mathbb {T}}^{2}\right)$. The limit in (24) then follows by the bounded convergence theorem.

## Appendix

In this “Appendix” we prove the a priori estimates used in the proof of existence of a solution for the Euler equation and we also review some fundamental results mentioned before. We start by introducing the Biot–Savart operator which establishes the connection between the velocity vector field *u* and the vorticity field $\omega$.

### Remark 21

(The Biot–Savart kernel) The vorticity field corresponding to a 2D incompressible fluid is conventionally regarded as a scalar quantity $\omega = curl \ u = \partial _2u_1 - \partial _1u_2$ (formally it is a vector $(0,0, \partial _2u_1 - \partial _1u_2)$ orthogonal to $u = (u_1, u_2, 0)$ [44]). It is known (see [44, 28]) that if $\psi : {\mathbb {T}}^2 \times [0, \infty ) \rightarrow {\mathbb {R}}$ is a solution for $\Delta \psi = -\omega$ then $u = \nabla ^{\perp }\psi$ solves $\omega = curl \ u$, so $u = -\nabla ^{\perp }\Delta ^{-1} \omega$. It is worth mentioning that the existence of a (unique, up to an additive constant) stream function $\psi$—and therefore the reconstruction of *u* from $\omega$ is ensured by the incompressibility condition $div \ u = 0$ [44]. A periodic, distributional solution of $\Delta \psi = -\omega$ is given by ( [28])

$$\begin{aligned} \psi (x) = (G \star \omega )(x) \end{aligned}$$

where *G* is the Green function of the operator $-\Delta$ on ${\mathbb {T}}^2$, $G(x) = \displaystyle \sum _{k \in {\mathbb {Z}}^2\setminus \{0\}} \frac{e^{ik \cdot x}}{\Vert k\Vert ^2}.$ Then the vector field $u=\nabla ^{\perp }\psi$ is uniquely derived from $\omega$ as follows:

25 $$\begin{aligned} u(x) = (K \star \omega )(x) = \displaystyle \int \limits _{{\mathbb {T}}^2} K(x-y)\omega (y)dy \end{aligned}$$

where *K* is the so-called *Biot–Savart kernel*

$$\begin{aligned} K(x) = \nabla ^{\perp } G(x) = \displaystyle \sum _{k \in {\mathbb {Z}}^2\setminus \{0\}} \frac{ik^{\perp }}{\Vert k\Vert ^2}e^{ik \cdot x} \end{aligned}$$

with $k=(k_1, k_2)$, $k^{\perp } = (k_2, -k_1)$. It is known that *G* is smooth everywhere except at $x=0$, and that $K \in L^1({\mathbb {T}}^2)$.

For the following result we recall a few elementary results of Fourier analysis. We embed $L^{2}\left( {\mathbb {T}}^{2}\right)$ into $L^{2}\left( {\mathbb {T}}^{2};{\mathbb {C}}\right)$ and consider the basis of functions $\left\{ e^{2\pi i\xi \cdot x};\xi \in {\mathbb {Z}}^{2}\right\}$. Then every $f\in L^{2}\left( {\mathbb {T}}^{2};{\mathbb {C}}\right)$ can be expressed as

$$\begin{aligned} f(x)=\sum _{\xi \in {\mathbb {Z}}^{2}}{\widehat{f}}\left( \xi \right) e^{2\pi i \xi \cdot x} \end{aligned}$$

where ${\widehat{f}}\left( \xi \right) =\int _{{\mathbb {T}}^{3}}e^{-2\pi i\xi \cdot x}f\left( x\right) dx$, $\xi \in {\mathbb {Z}}^{2}$ are the corresponding Fourier coefficients. We have the classical Parseval identity (see e.g. [44])

$$\begin{aligned} \int \limits _{{\mathbb {T}}^{2}}\left| f\left( x\right) \right| ^{2}dx=\sum _{\xi \in {\mathbb {Z}}^{2}}\left| {\widehat{f}}\left( \xi \right) \right| ^{2}. \end{aligned}$$

If $v\in L^{2}\left( {\mathbb {T}}^{2};{\mathbb {R}}^{2}\right)$ is a vector field with components $v_{i}$, $i=1,2$, we write ${\widehat{v}}\left( \xi \right) =\int _{{\mathbb {T}}^{2}}e^{2\pi i\xi \cdot x}v\left( x\right) dx$ and we have, in a similar way, that $\int _{{\mathbb {T}}^{2}}\left| v\left( x\right) \right| ^{2}dx=\sum _{\xi \in {\mathbb {Z}}^{2}}\left| {\widehat{v}}\left( \xi \right) \right| ^{2}$. Since *u* and $\omega$ are partial derivatives of other functions on the torus, they must have zero average:

$$\begin{aligned} \int \limits _{{\mathbb {T}}^{2}}u^{1}\left( x\right) dx=\int \limits _{{\mathbb {T}}^{2}}u^{2}\left( x\right) dx=\int \limits _{{\mathbb {T}}^{2}}\omega \left( x\right) dx=0. \end{aligned}$$

Hence $\widehat{u^{1}}\left( 0,0\right) =\widehat{u^{2}}\left( 0,0\right) ={\widehat{\omega }}\left( 0,0\right) =0$ and the term corresponding to $\xi =(0,0)$ does not appear in the Fourier expansion for $u_1$, $u_2$ and, respectively $\omega$.

For every $s\ge 0$, let $W^{s,2}\left( {\mathbb {T}}^{2};{\mathbb {C}}\right)$ be the fractional Sobolev space of all functions $f\in L^{2}\left( {\mathbb {T}} ^{2};{\mathbb {C}}\right)$ such that

$$\begin{aligned} \sum _{\xi \in {\mathbb {Z}}^{2} }\left| \xi \right| ^{2s}\left| {\widehat{f}}\left( \xi \right) \right| ^{2}<\infty . \end{aligned}$$

It is a simple exercise to show that there exist a constant $C>1$ such that if $s\in {\mathbb {N}}$ the

$$\begin{aligned} C^{-1}\left\| f\right\| _{s,2}^{2}\le \sum _{\xi \in {\mathbb {Z}}^{2} }(1+\left| \xi \right| ^{2s})\left| {\widehat{f}}\left( \xi \right) \right| ^{2}<C\left\| f\right\| _{s,2}^{2} \end{aligned}$$

It follows that this definition coincides with the definition given in Sect. 2 for integer $s\in {\mathbb {N}}$. Therefore we can extend the norm $\left\| f\right\| _{s,2}$ defined for $s\in {\mathbb {N}}$ to arbitrary $s>0$ to be given by

$$\begin{aligned} \left\| f\right\| _{s,2}^{2}= \sum _{\xi \in {\mathbb {Z}}^{2} }(1+\left| \xi \right| ^{2s})\left| {\widehat{f}}\left( \xi \right) \right| ^{2} \end{aligned}$$

We denote by $W_{\sigma }^{s,2}\left( {\mathbb {T}} ^{2},{\mathbb {R}}^{2}\right)$ the space of all zero mean divergence free (divergence in the sense of distribution) vector fields $v\in L^{2}\left( {\mathbb {T}}^{2};{\mathbb {R}}^{2}\right)$ such that all components $v_{i}$, $i=1,2$ belong to $W^{s,2}\left( {\mathbb {T}}^{2};{\mathbb {R}}\right)$. For a vector field $v\in W_{\sigma }^{s,2}\left( {\mathbb {T}}^{2},{\mathbb {R}} ^{2}\right)$ the norm $\left\| v\right\| _{s,2}$ is defined by the identity $\left\| v\right\| _{s,2}^{2}=\sum _{i=1} ^{2}\left\| v_{i}\right\| _{s,2}^{2}$, where $\left\| v_{i}\right\| _{s,2}^{2}$ is defined above. We thus have again $\left\| v\right\| _{s,2}^{2}:=\sum _{\xi \in {\mathbb {Z}} ^{2}\backslash \left\{ 0\right\} }\left| \xi \right| ^{2s}(\left| {\widehat{v}}_1\left( \xi \right) \right| + \left| {\widehat{v}}_1\left( \xi \right) \right| )^{2}$. For $f\in W^{s,2}\left( {\mathbb {T}}^{2};{\mathbb {C}}\right)$, we denote by $\left( -\Delta \right) ^{s/2}f$ the function of $L^{2}\left( {\mathbb {T}}^{3};{\mathbb {C}}\right)$ with Fourier coefficients $\left| \xi \right| ^{s}{\widehat{f}}\left( \xi \right)$. For even integers $s\in {\mathbb {N}}$ , this definition coincides with the classical definition. Similarly, we write $-\Delta ^{-1}f$ for the function having Fourier coefficients $\left| \xi \right| ^{-2}{\widehat{f}}\left( \xi \right)$. We use the same notations for vector fields, meaning that the operations are made componentwise.

The Biot–Savart operator is the reconstruction of a zero mean divergence free vector field *u* from a divergence free vector field $\omega$ such that ${\text {curl}}u=\omega$. As stated in Remark  21 on the 2D torus it is given by $u=-{\text {curl}}\Delta ^{-1}\omega$. It follows that the Fourier coefficients of *u* are given by ${\widehat{u}}\left( \xi \right) =\left| \xi \right| ^{-2}\xi ^{\perp } {\widehat{\omega }}\left( \xi \right)$, where $\xi ^{\perp }=(\xi _1,\xi _2)^{\perp }=(\xi _2,-\xi _1)$.

In the next proposition we highlight the smoothing properties of the Biot–Savart kernel *K*.

### Proposition 22

(The Biot–Savart law, [18, 44]) Let *u* be the divergence-free, zero average, vector field defined as $u = -curl \Delta ^{-1} \omega$. Then, for any $s\ge 0$, there exists a constant $C_{s,2}$, independent of *u* such that

26 $$\begin{aligned} \Vert u\Vert _{s+1, 2} \le C_{s,2}\Vert \omega \Vert _{s,2}. \end{aligned}$$

### Proof

Using the definition given above of $\left\| u\right\| _{s+1,2}^{2}$, the formula which relates ${\widehat{u}}\left( \xi \right)$ and ${\widehat{\omega }}\left( \xi \right)$ and the rule $\left| a\times b\right| \le \left| a\right| \left| b\right|$, we get

$$\begin{aligned} \left\| u\right\| _{s+1,2}^{2}= & {} \sum _{\xi \in {\mathbb {Z}} ^{2}\backslash \left\{ 0\right\} }(1+\left| \xi \right| ^{2s+2})\left| {\widehat{u}}\left( \xi \right) \right| ^{2}\\\le & {} \sum _{\xi \in {\mathbb {Z}} ^{2}\backslash \left\{ 0\right\} }(1+\left| \xi \right| ^{2s+2})\left| \xi \right| ^{-4}\vert \xi ^\perp {\widehat{\omega }}\left( \xi \right) \vert ^{2}\\\le & {} \sum _{\xi \in {\mathbb {Z}}^{2}\backslash \left\{ 0\right\} }(\left| \xi \right| ^{-2}+\left| \xi \right| ^{2s})\left| {\widehat{\omega }}\left( \xi \right) \right| ^{2}\\\le & {} \sum _{\xi \in {\mathbb {Z}}^{2}\backslash \left\{ 0\right\} }(1+\left| \xi \right| ^{2s})\left| {\widehat{\omega }}\left( \xi \right) \right| ^{2} \end{aligned}$$

and the latter is precisely equal to $\left\| \omega \right\| _{s,2}$. $\square$

### Remark 23

The norm $\Vert \cdot \Vert _{m,2}$ is equivalent to the norm defined as $||| f |||:= \Vert f\Vert _2 + \Vert D^{m}f\Vert _2$, therefore it is enough to show that all properties hold for the $L^2$ norm of *f* and for the $L^2$ norm of the maximal derivative $D^mf$ (see [14] pp. 217).

Let $\omega$ be the solution of the Euler Eq. (1) and $\omega ^{\nu _n,R,n}$ the solution of the linear approximating Eq. (12). In the following lemmas we collect a number of identities and a priori estimates. Lemma 24 in particular in proving that the solution of the Euler equation is global.

### Lemma 24

There exists a constant ${\mathcal {C}}={\mathcal {C}}(\omega _0,T)$ which is independent of the truncation radius *R* such that

$$\begin{aligned} {\mathbb {E}}\left[ \displaystyle \sup _{s \in [0,t]} \left( \ln (e + \Vert \omega _s\Vert _{k,2}^2)\right) \right] \le {\mathcal {C}}(\Vert \omega _0\Vert _{k,2},T). \end{aligned}$$

### Proof

By the Biot–Savart law

$$\begin{aligned} \begin{aligned} u(x) = \displaystyle \int \limits _{{\mathbb {T}}^2} K(x-y)\omega (y)dy = - \frac{1}{4\pi } \displaystyle \int \limits _{{\mathbb {T}}^2} \frac{(x-y)^{\perp }}{|x-y|^2} \omega (y)dy \end{aligned} \end{aligned}$$

We use the truncation

$$\begin{aligned} z_{\epsilon }(x) = {\left\{ \begin{array}{ll} 1, &{} \text {if }|x|\le \epsilon \\ 0, &{} \text {if }|x| > 2\epsilon \end{array}\right. } \end{aligned}$$

with $$|\nabla z_{\epsilon }(x)| \le \frac{C}{\epsilon }, \epsilon \le 1$$. Let

$$\begin{aligned} u^1(x):= & {} -\frac{1}{4 \pi } \displaystyle \int \limits _{{\mathbb {T}}^2} z_{\rho }(x-y)K(x-y)\omega (y)dy \\ u^2(x):= & {} -\frac{1}{4 \pi } \displaystyle \int \limits _{{\mathbb {T}}^2}\left( 1- z_{\epsilon }(x-y)\right) K(x-y)\omega (y)dy. \end{aligned}$$

Then

$$\begin{aligned} \nabla u^1(x)&:= - \frac{1}{4\pi } \displaystyle \int \limits _{{\mathbb {T}}^2} z_{\epsilon }(x-y)K(x-y)\nabla \omega (y) dy \\&\le \left( \displaystyle \int \limits _{{\mathbb {T}}^2} \left( z_{\epsilon }(x-y)K(x-y)\right) ^{4/3}\right) ^{3/4} \left( \displaystyle \int \limits _{{\mathbb {T}}^2} \left( \nabla \omega (y)\right) ^4dy\right) ^{1/4} \\&\le \Vert K\Vert _{L^{4/3}(\{y:|x-y|< 2\epsilon \})} \Vert \nabla \omega \Vert _{L^{4}(\{y:|x-y| < 2\epsilon \})} \\&\le C \epsilon ^{2/3} \Vert \nabla \omega \Vert _{4} \\&\le C \epsilon ^{2/3} \Vert u\Vert _{3,2} \end{aligned}$$

given that $\Vert \nabla \omega \Vert _4 \le C \Vert \nabla \omega \Vert _{1,2} \le C\Vert \omega \Vert _{2,2} \le C \Vert u\Vert _{3,2}$ by the Sobolev embedding $W^{1,2} \hookrightarrow L^4$ and the Biot–Savart law. So

$$\begin{aligned} \Vert \nabla u^1(x)\Vert _{\infty } \le C \epsilon ^{2/3} \Vert u\Vert _{3,2}. \end{aligned}$$

For the second integral

$$\begin{aligned} \begin{aligned} \nabla u^2(x) := - \frac{1}{4\pi } \displaystyle \int \limits _{{\mathbb {T}}^2} \nabla \left( (1 - z_{\epsilon }(x-y))K(x-y)\right) \omega (y)dy \end{aligned} \end{aligned}$$

we use the fact that

$$\begin{aligned} |\nabla K(x-y)| \le \frac{C}{|x-y|^2} \end{aligned}$$

so

$$\begin{aligned} \Vert \nabla u^2(x)\Vert _{\infty } \le C \left( 1 - \ln \epsilon \right) \Vert \omega \Vert _{\infty }. \end{aligned}$$

That is

$$\begin{aligned} \Vert \nabla u\Vert _{\infty } \le C \left( \epsilon ^{2/3}\Vert u\Vert _{3,2} + (1- \ln \epsilon ) \Vert \omega \Vert _{\infty }\right) . \end{aligned}$$

If $\Vert u\Vert _{3,2} \le 1$ we choose $\epsilon = 1$, otherwise we choose $\epsilon = \frac{1}{\Vert u\Vert _{3,2}^{3/2}}$. We have

$$\begin{aligned} \Vert \nabla u\Vert _{\infty } \le C \left( 1 + \left( 1 + \frac{3}{2} \ln \Vert u\Vert _{3,2}\right) \Vert \omega \Vert _{\infty }\right) \end{aligned}$$

Using the inequality $1 + \ln ^{+} x \le C \ln (e+x)$ for sufficiently large *C*, we have

$$\begin{aligned} \begin{aligned} \Vert \nabla u_t\Vert _{\infty }&\le C \left( 1 + \left( 1 + \ln ^{+}\Vert u_t\Vert _{3,2}\right) \Vert \omega _t\Vert _{\infty }\right) \\&\le C \left( 1 + \ln ( e + \Vert u_t\Vert _{3,2})\Vert \omega _t\Vert _{\infty }\right) \end{aligned} \end{aligned}$$

and using the transport property and Biot–Savart $\Vert u_t\Vert _{3,2} \le \Vert \omega _t\Vert _{2,2} \le \Vert \omega _t\Vert _{2,2}$ we have

27 $$\begin{aligned} \begin{aligned} \Vert \nabla u_t\Vert _{\infty }&\le C \left( 1 + \ln ( e + \Vert \omega _t\Vert _{k,2})\Vert \omega _0\Vert _{\infty }\right) \end{aligned} \end{aligned}$$

By the Itô formula

where

Define

$$\begin{aligned} Z_t : = \displaystyle \int \limits _0^t (1 + \Vert \omega _s\Vert _{\infty }) ds. \end{aligned}$$

We have

28 $$\begin{aligned} e^{-C_2 Z_t} \ln (e + \Vert \omega _t\Vert _{k,2}^2) \le \ln (e + \Vert \omega _0\Vert _{k,2}^2) + \displaystyle \int \limits _0^t e^{-C_2Z_s} dY_s. \end{aligned}$$

By the Burkholder–Davis–Gundy inequality

$$\begin{aligned} {\mathbb {E}}\left[ \displaystyle \sup _{s \in [0,t]} \left| \displaystyle \int \limits _0^s dY_r\right| \right] \le \alpha {\mathbb {E}}\left[ \left\langle \displaystyle \int \limits _0^{\cdot } dY_r \right\rangle _t^{1/2} \right] \end{aligned}$$

We control the quadratic variation of the stochastic integral using the fact that

$$\begin{aligned} \displaystyle \sum _{i=1}^{\infty }\Vert \xi _i\Vert _{3,2} < \infty \end{aligned}$$

and

since

and

We write

In consequence, the Burkholder–Davis–Gundy inequality gives

$$\begin{aligned} {\mathbb {E}}\left[ \displaystyle \sup _{s\in [0,t]} \left| \displaystyle \int \limits _0^s dY_r\right| \right] \le C \sqrt{t}. \end{aligned}$$

Using this in 28 we obtain

$$\begin{aligned} {\mathbb {E}}\left[ \displaystyle \sup _{s \in [0,t]} \left( e^{-C\displaystyle \int \limits _0^s(1+\Vert \omega _r^R\Vert _{\infty })dr} \ln (e + \Vert \omega _s^R\Vert _{k,2}^2)\right) \right] \le \ln (e + \Vert \omega _0\Vert _{k,2}^2) + C\sqrt{t}. \end{aligned}$$

By the Fatou lemma this holds also for the original solution $\omega$

$$\begin{aligned} \begin{aligned}&{\mathbb {E}}\left[ \displaystyle \sup _{s \in [0,t]} \left( e^{-C\displaystyle \int \limits _0^s(1+\Vert \omega _r\Vert _{\infty })dr} \ln (e + \Vert \omega _s\Vert _{k,2}^2)\right) \right] \\&\quad \le {\mathbb {E}}\left[ \displaystyle \sup _{s \in [0,t]} \displaystyle \lim \inf _{R \rightarrow \infty }\left( e^{-C\displaystyle \int \limits _0^s(1+\Vert \omega _r^R\Vert _{\infty })dr} \ln (e + \Vert \omega _s^R\Vert _{k,2}^2)\right) \right] \\&\quad \le \ln (e + \Vert \omega _0\Vert _{k,2}^2) + C\sqrt{t}.\\ \end{aligned} \end{aligned}$$

Using the transport property (see relation (22) in Sect. 7), $\Vert \omega _t\Vert _{\infty } = \Vert \omega _0\Vert _{\infty }$, we conclude that there exists a constant ${\mathcal {C}}(\Vert \omega _0\Vert _{k,2},T)$ which is independent of the truncation radius *R* such that

$$\begin{aligned} {\mathbb {E}}\left[ \displaystyle \sup _{s \in [0,t]} \left( \ln (e + \Vert \omega _s\Vert _{k,2}^2)\right) \right] \le {\mathcal {C}}(\Vert \omega _0\Vert _{k,2},T). \end{aligned}$$

$$\square $$

### Lemma 25

The following properties hold:

i.: For any $f\in {\mathcal {W}}^{2,2}({\mathbb {T}}^2)$ we have
ii.: If $\omega _t, \omega _t^{\nu _n,R,n}\in {\mathcal {W}}^{k,2}({\mathbb {T}}^2)$ then the following $L^2$-transport formulae^16^ hold ${\mathbb {P}}$-almost surely: $$\begin{aligned} \Vert \omega _t\Vert _{L^2} =\Vert \omega _0\Vert _{L^2} \ \ \ \mathrm {and}\ \ \ \ \Vert \omega _t^{\nu _n,R,n}\Vert _{L^2}\le \Vert \omega _0\Vert _{L^2}. \end{aligned}$$
iii.: If $\omega \in {\mathcal {W}}^{k,2}({\mathbb {T}}^2)$, then $(P_t^{n-1,n})_t$ defined in (14) and are processes with paths taking values in $L^2({\mathbb {T}}^2)$.
iv.: There exists a constant $C_1$ such that:
v.: There exist some constants $C_2$ and $C_2^{\prime }$ such that: with $0<a\le 1$, and
vi.: For arbitrary $p\ge 2$, there exists a constant $C_p(T)$ independent of *n* such that $$\begin{aligned} {\mathbb {E}}\big [\displaystyle \sup _{t \in [0,T]}\Vert \omega _t^{\nu _n,R,n}\Vert _{k,2}^p\big ] \le C_p(T). \end{aligned}$$

### Proof of Lemma 25

i. Since the dual of  is  by Remark 2, observe that
  This is an intrinsic property of the operator , which holds even when *f* is not a solution of the Euler equation or of the approximating sequence.
ii. By the Itô formula
  The last sum has been proved to be 0 at i. and  by integration by parts since $u_t^{\nu _{n-1},R,n-1}$ is divergence-free. Similarly  since the vector fields $(\xi _i)_i$ are assumed to be divergence-free. Therefore
  $$\begin{aligned} \Vert \omega _t^{\nu _n,R,n}\Vert _2 = \Vert \omega _0\Vert _2^2 - 2\displaystyle \int \limits _0^t \nu _n (\nabla \omega _s^{\nu _n,R,n})^2ds \le \Vert \omega _0\Vert _2^2 \ \ \ \ \ \ {\mathbb {P}}\hbox { - almost surely}. \end{aligned}$$
  The calculations are similar for the Euler equation, but there are no viscous terms, hence
  $$\begin{aligned} \Vert \omega _t\Vert _2 = \Vert \omega _0\Vert _2^2 \ \ \ \ \ \ {\mathbb {P}}\hbox { - almost surely}. \end{aligned}$$
iii. One has
  $$\begin{aligned} \Vert u_t^{\nu _{n-1},R,n-1} \cdot \nabla \omega _t^{\nu _n,R,n}\Vert _{2}^2 \le C\Vert u_t^{\nu _{n-1},R,n-1}\Vert _{\infty }^2 \Vert \nabla \omega _t^{\nu _n,R,n}\Vert _2^2 \le C\Vert \omega _t^{\nu _n,R,n}\Vert _{k,2}^4 \end{aligned}$$
  due to Hölder’s inequality and the following inequalities:
  - $\Vert u_t^{\nu _{n-1},R,n-1}\Vert _{\infty } \le C \Vert \nabla u_t^{\nu _{n-1},R,n-1}\Vert _{\infty }$ by Poincaré’s inequality (or using directly $\Vert u_t^{\nu _{n-1},R,n-1}\Vert _{\infty } \le \Vert u_t^{\nu _{n-1},R,n-1}\Vert _{k,2} \le \Vert \omega _t^{\nu _{n-1},R,n-1}\Vert _{k-1,2}$)
  - $\Vert \nabla u_t^{\nu _{n-1},R,n-1}\Vert _{\infty } \le C \Vert \nabla u_t^{\nu _{n-1},R,n-1}\Vert _{k,2}$ by the Sobolev embedding theorem ${\mathcal {W}}^{k,2}({\mathbb {T}}^2) \hookrightarrow L^{\infty }({\mathbb {T}}^2)$ ( [1], Theorem 4.12, case A, $m=k, p=n=2, q= \infty$).
  - $\Vert \nabla u_t^{\nu _{n-1},R,n-1}\Vert _{k,2} \le \Vert u_t^{\nu _{n-1},R,n-1}\Vert _{k+1,2}$ by the definition of the Sobolev norm
  - $\Vert u_t^{\nu _{n-1,R,n-1}}\Vert _{k+1,2} \le C\Vert \omega _t^{\nu _{n-1},R,n-1}\Vert _{k,2}$ by the Biot–Savart law.
  All the other terms which involve $\xi _i$ stay in $L^2$ according to the initial Assumptions 4, therefore the conclusion holds also for the process .
iv. Let us denote $\omega _t^{\nu _n,R,n}$ shortly by $\omega _t^{\nu }$. We have to estimate
  Remark that
  where . One can write
  Due to our initial assumptions on the vector fields $(\xi _i)_i$, each term can be bounded by $\Vert \omega _t^{\nu }\Vert _{k,2}^2$, so the required inequality is proven.
v. For $\beta \ge 2$ we have
  Using Hölder and Cauchy-Schwartz inequalities one has
  $$\begin{aligned} \begin{aligned}&\displaystyle \int \limits _{{\mathbb {T}}^2} |\partial ^k \omega _t^{\nu _n,R,n} \cdot \partial ^{\beta }u_t^{\nu _{n-1},R,n-1} \cdot \partial ^{k-\beta }(\nabla \omega _t^{\nu _n,R,n})|dx\\&\quad \le \Vert \partial ^k\omega _t^{\nu _n,R,n}\Vert _2\Vert \partial ^{\beta }u_t^{\nu _{n-1},R,n-1}\cdot \partial ^{k-\beta }(\nabla \omega _t^{\nu _n,R,n})\Vert _2 \\&\quad \le \Vert \partial ^k\omega _t^{\nu _n,R,n}\Vert _2\Vert \partial ^{\beta }u_t^{\nu _{n-1},R,n-1}\Vert _4\Vert \partial ^{k- \beta }(\nabla \omega _t^{\nu _n,R,n})\Vert _4 \\&\quad \le \Vert \omega _t^{\nu _n,R,n}\Vert _{k,2}\Vert \partial ^{\beta }u_t^{\nu _{n-1},R,n-1}\Vert _4\Vert \partial ^{k-\beta }( \nabla \omega _t^{\nu _n,R,n})\Vert _4 \end{aligned} \end{aligned}$$
  By Gagliardo–Nirenberg inequality
  $$\begin{aligned} \begin{aligned} \Vert \partial ^{\beta }u_t^{\nu _{n-1},R,n-1}\Vert _4&\le C\Vert \partial ^{\beta +1}u_t^{\nu _{n-1},R,n-1}\Vert _2^a\Vert u_t^{\nu _{n-1},R,n-1}\Vert _2^{1-a} \\&\le C\Vert u_t^{\nu _{n-1},R,n-1}\Vert _{\beta +1,2}^a\Vert u_t^{\nu _{n-1},R,n-1}\Vert _2^{1-a} \\&\le C \Vert u_t^{\nu _{n-1},R,n-1}\Vert _{k+1,2}^a\Vert u_t^{\nu _{n-1},R,n-1}\Vert _2^{1-a} \\&\le C \Vert \omega _t^{\nu _{n-1},R,n-1}\Vert _{k,2}^a\Vert u_t^{\nu _{n-1},R,n-1}\Vert _2^{1-a} \\&\le C \Vert \omega _t^{\nu _{n-1},R,n-1}\Vert _{k,2} \end{aligned} \end{aligned}$$
  since $\Vert u_t^{\nu _{n-1},R,n-1}\Vert _2^{1-a} \le \Vert u_t^{\nu _{n-1},R,n-1}\Vert _{2,2}^{1-a} \le \Vert u_t^{\nu _{n-1},R,n-1}\Vert _{k+1,2}^{1-a} \le C\Vert \omega _t^{\nu _{n-1},R,n-1}\Vert _{k,2}^{1-a}$ by the Biot–Savart law. Similarly
  $$\begin{aligned} \begin{aligned} \Vert \partial ^{k-\beta }(\nabla \omega _t^{\nu _n,R,n})\Vert _4&\le C\Vert \partial ^{k-\beta +1}(\nabla \omega _t^{\nu _n,R,n})\Vert _2^a \Vert \nabla \omega _t^{\nu _n,R,n}\Vert _2^{1-a} \\&\le C\Vert \nabla \omega _t^{\nu _n,R,n}\Vert _{k-\beta +1,2}^a \Vert \omega _t^{\nu _n,R,n}\Vert _{2,2}^{1-a} \\&\le C\Vert \omega _t^{\nu _n,R,n}\Vert _{k,2}^a\Vert \omega _t^{\nu _n,R,n}\Vert _{k,2}^{1-a} \\&= C\Vert \omega _t^{\nu _n,R,n}\Vert _{k,2} \end{aligned} \end{aligned}$$
  Therefore
  For $$\beta = 0:$$
  $$\begin{aligned} \begin{aligned}&\displaystyle \int \limits _{{\mathbb {T}}^2}\partial ^k\omega _t^{\nu _n,R,n} \cdot u_t^{\nu _{n-1},R,n-1}\cdot \partial ^k(\nabla \omega _t^{\nu _n,R,n})dx \\&\quad = \displaystyle \int \limits _{{\mathbb {T}}^2} u_t^{\nu _{n-1},R,n-1}\cdot \nabla (\partial ^k\omega _t^{\nu _n,R,n}) \cdot \partial ^k\omega _t^{\nu _n,R,n}dx \\&\quad = \frac{1}{2}\displaystyle \int \limits _{{\mathbb {T}}^2} u_t^{\nu _{n-1},R,n-1} \cdot \nabla |\partial ^k\omega _t^{\nu _n,R,n}|^2dx\\&\quad = -\frac{1}{2}\displaystyle \int \limits _{{\mathbb {T}}^2} (\partial ^k\omega _t^{\nu _n,R,n})^2(\nabla \cdot u_t^{\nu _{n-1},R,n-1})dx \\&\quad = 0. \end{aligned} \end{aligned}$$
  For $$\beta = 1:$$
  $$\begin{aligned} \begin{aligned} \Vert \nabla u_t^{\nu _{n-1},R,n-1}\cdot \partial ^k\omega _t^{\nu _n,R,n}\Vert _2&\le \Vert \nabla u_t^{\nu _{n-1},R,n-1}\Vert _{\infty }^2\Vert \partial ^k\omega _t^{\nu _n,R,n}\Vert _2 \\&\le C\Vert \nabla u_t^{\nu _{n-1},R,n-1}\Vert _{k,2} \Vert \omega _t^{\nu _n,R,n}\Vert _{k,2} \\&\le C\Vert u_t^{\nu _{n-1},R,n-1}\Vert _{k+1,2} \Vert \omega _t^{\nu _n,R,n}\Vert _{k,2} \\&\le C\Vert \omega _t^{\nu _{n-1},R,n-1}\Vert _{k,2}\Vert \omega _t^{\nu _n,R,n}\Vert _{k,2}. \end{aligned} \end{aligned}$$
  We used the embedding ${\mathcal {W}}^{k,2} \hookrightarrow L^{\infty }$ and the Biot–Savart law. We need this property for relative compactness (see Proposition 17).
vi. After applying the Itô formula we obtain
  We analyse each term. One can write
  $$\begin{aligned} \langle \partial ^k\omega _s^{\nu _n,R,n},\partial ^{k+2}\omega _s^{\nu _n,R,n}\rangle = -\Vert \partial ^{k+1}\omega _s^{\nu _n,R,n}\Vert _2^2 \le 0. \end{aligned}$$
  We want to estimate the other terms independently of $\nu _n$. All terms are estimated above. Let
  Obviously $B_t$ is a local martingale. Using the Burkholder–Davis–Gundy inequality there exists a constant $\tilde{\alpha }_p$ such that
  $$\begin{aligned} {\mathbb {E}}\big [\displaystyle \sup _{s\in [0,t]}|B_s|^p\big ] \le \tilde{\alpha }_p {\mathbb {E}}\left[ \langle B\rangle _t^{\frac{p}{2}}\right] . \end{aligned}$$
  Then
  $$\begin{aligned} \displaystyle {\mathbb {E}}\big [\langle B\rangle _t^{\frac{p}{2}}\big ] \le C_{p,T}^2\int \limits _{0}^{t}{\mathbb {E}}[\displaystyle \sup _{r\in [0,s]} \beta _r^p]ds \end{aligned}$$
  following the same calculations as those from step v. and taking into account Assumption (4). We have, for $t\in [0,T]$,
  $$\begin{aligned} \beta _t\le & {} \beta _0 -2B_t+ (C_2'+C_1) \displaystyle \int \limits _0^t\beta _sds \\ \sup _{s\in [0,t]}\beta _s^p\le & {} 3^{p-1} \beta _0^p + 2\times 3^{p-1} \sup _{s\in [0,t]}| B_s|^p+ 3^{p-1}T(C_2'+C_1)\displaystyle \int \limits _0^t\sup _{r\in [0,s]}\beta _r^{p}ds. \end{aligned}$$
  In conclusion
  $$\begin{aligned} {\mathbb {E}}\big [\displaystyle \sup _{s\in [0,t]} \beta _t^p\big ] \le \tilde{C_1}(T)\beta _0^p + \tilde{C_2}(T)\displaystyle \int \limits _0^t {\mathbb {E}}\big [\displaystyle \sup _{s\in [0,t]} \beta _s^p]ds. \end{aligned}$$
  Finally, using Gronwall’s inequality, we deduce that
  $$\begin{aligned} {\mathbb {E}}\big [\displaystyle \sup _{s\in [0,t]}\Vert \omega _s^{\nu _n,R,n}\Vert _{k,2}^p\big ] \le C_p(T) \end{aligned}$$
  with $$C(T):= {\tilde{C}}_1(T)\beta _0^p\exp ({\tilde{C}}_2(T)T)$$. $$\square $$

The following lemma is instrumental in showing that the limit of the approximating sequence satisfies the Euler equation in ${\mathcal {W}}^{k,2}({\mathbb {T}}^2)$ although the relative compactness property holds in $D\big ([0,T], L^{2}({\mathbb {T}}^2)\big )$. It is also essential when proving a priori estimates for the truncated solution $\omega ^R$ (see Lemma 27).

### Lemma 26

i. Assume that $(a_n)_n$ is a sequence of functions such that $\displaystyle \lim \nolimits _{n\mapsto \infty } a_n=a$ in $L^2({\mathbb {T}}^2)$ and $\displaystyle \sup \nolimits _{n>1} \left\| a_n\right\| _{s,2}<\infty$ for $s \ge 0$. Then $a \in {\mathcal {W}}^{s,2}({\mathbb {T}}^2)$ and $\left\| a \right\| _{s,2}<\displaystyle \sup \nolimits _{n>1}\left\| a_n\right\| _{s,2}$. Moreover, $\displaystyle \lim \nolimits _{n\mapsto \infty }a_n = a$ in ${\mathcal {W}}^{s',2}({\mathbb {T}}^2)$ for any $s'<s$.
ii. Assume that $a_n:\Omega \mapsto {\mathcal {W}}^{s,2}({\mathbb {T}}^2)$ is a sequence of measurable maps such that, $\displaystyle \lim \nolimits _{n\mapsto \infty } a_n=a$ in $L^2({\mathbb {T}}^2)$, ${\mathbb {P}}$-almost surely or $\displaystyle \lim \nolimits _{n\mapsto \infty } a_n=a$ in distribution. Further assume that $\displaystyle \sup \nolimits _{n>1}{\mathbb {E}}[\left\| a_n\right\| _{s,2}^{p}]<\infty$. Then, ${\mathbb {P}}$-almost surely, $a\in {\mathcal {W}}^{s,2}({\mathbb {T}}^2)$ and ${\mathbb {E}}[\left\| a\right\| _{s,2}^{p}] \le \displaystyle \sup \nolimits _{n>1}{\mathbb {E}}[\left\| a_n\right\| _{s,2}^{p}]$, for any $p>0$.

### Proof of Lemma 26

i. Since $\displaystyle \lim \nolimits _{n \rightarrow \infty } a_n = a$ in $L^2({\mathbb {T}}^2)$ it follows that for arbitrary $\lambda \in {\mathbb {Z}}^2$ we have
  $$\begin{aligned} \lim _{n\mapsto \infty } \widehat{a_n}\left( \lambda \right) =\lim _{n\mapsto \infty } \int \limits _{{\mathbb {T}}^{2}}e^{2\pi i\lambda \cdot x} a_n\left( x\right) dx=\int \limits _{{\mathbb {T}}^{2}}e^{2\pi i\lambda \cdot x} a\left( x\right) dx={\widehat{a}}(\lambda ) \end{aligned}$$
  Therefore by Fatou’s lemma
  $$\begin{aligned} \left\| a\right\| _{s,2}^{2}= & {} \sum _{\lambda \in {\mathbb {Z}}^{2} }(1+\left| \lambda \right| ^{2s})\left| {\widehat{a}}\left( \lambda \right) \right| ^{2}= \sum _{\lambda \in {\mathbb {Z}}^{2} }(1+\left| \lambda \right| ^{2s})\liminf _{n\mapsto \infty }\left| \widehat{a_n}\left( \lambda \right) \right| ^{2}\\\le & {} \liminf _{n\mapsto \infty }\sum _{\lambda \in {\mathbb {Z}}^{2} }(1+\left| \lambda \right| ^{2s})\left| \widehat{a_{n}}\left( \lambda \right) \right| ^{2}\\= & {} \liminf _{n\mapsto \infty }\left\| a_n\right\| _{s,2}^{2}\le \sup _{n\ge 1}\left\| a_n\right\| _{s,2}^2. \end{aligned}$$
  For the second part we can write
  $$\begin{aligned} \Vert a_n-a\Vert _{s',2}= & {} \displaystyle \sum _{\lambda \in {\mathbb {Z}}^2, |\lambda |\le M} (1+|\lambda |^{2s'})|({\hat{a}}_n - {\hat{a}})\lambda | \\&+ \displaystyle \sum _{\lambda \in {\mathbb {Z}}^2, |\lambda |\ge M} (1+|\lambda |^{2s'})|({\hat{a}}_n - {\hat{a}})\lambda |. \end{aligned}$$
  Note that
  $$\begin{aligned} \begin{aligned} \displaystyle \sum _{\lambda \in {\mathbb {Z}}^2, |\lambda |\ge M} (1+|\lambda |^{2s'})|({\hat{a}}_n - {\hat{a}})\lambda |&\le \displaystyle \sum _{\lambda \in {\mathbb {Z}}^2, |\lambda |\ge M} \frac{3(1+|\lambda |^{2s})}{1+M^{2(s-s')}}|({\hat{a}}_n - {\hat{a}})\lambda | \\&\le \frac{3}{1+M^{2(s-s')}}\big (\displaystyle \sup _{n\ge 1}\Vert a_n\Vert _{s,2}^2 + \Vert a\Vert _{s,2}^2\big ) \end{aligned} \end{aligned}$$
  where the first inequality is true due to the fact that $|\lambda |\ge M$. Now we can choose *M* such that the last term is strictly smaller than $\frac{\epsilon }{2}$. Likewise, *n* can be chosen such that
  $$\begin{aligned} \displaystyle \sum _{\lambda \in {\mathbb {Z}}^2, |\lambda |\le M} (1+|\lambda |^{2s'})|({\hat{a}}_n - {\hat{a}})\lambda | < \frac{\epsilon }{2} \end{aligned}$$
  hence $a_n$ converges to *a* in ${\mathcal {W}}^{s',2}$.
ii. From above it follows that
  $$\begin{aligned} \left\| a\right\| _{s,2}^{p} =(\left\| a\right\| _{s,2}^{2})^\frac{p}{2} \le ( \liminf _{n\ge 1}\left\| a_n\right\| _{s,2}^2)^\frac{p}{2}= \liminf _{n\ge 1}\left\| a_n\right\| _{s,2}^p \end{aligned}$$
  and therefore
  $$\begin{aligned} {\mathbb {E}} [\left\| a\right\| _{s,2}^{p}]\le \liminf _{n\mapsto \infty }{\mathbb {E}} [\left\| a_n\right\| _{s,2}^{p}]\le \sup _{n\ge 1}{\mathbb {E}} [\left\| a_n\right\| _{s,2}^p]. \end{aligned}$$
  $$\square $$

### Lemma 27

Let $\omega _t^R$ be the solution of the truncated Euler equation (11). There exists a constant ${\tilde{C}}(T)$ independent of *R* such that

$$\begin{aligned} {\mathbb {E}}\big [\displaystyle \sup _{t \in [0,T]}\Vert \omega _t^{R}\Vert _{k,2}^4\big ] \le {\tilde{C}}(T). \end{aligned}$$

### Proof

We deduce that there exists a constant ${\hat{C}}(T)$ such that

29 $$\begin{aligned} \sup _{t \in [0,T]}{\mathbb {E}}\big [\displaystyle \Vert \omega _t^{R}\Vert _{k,2}^4\big ] \le {\hat{C}}(T). \end{aligned}$$

from Lemma 26ii. with

$$\begin{aligned} a_n := a_{n,t}:=\displaystyle \omega _t^{\nu _n,R,n} \ \ \ \hbox {and} \ \ \ a:=a_t:=\displaystyle \omega _t^R. \end{aligned}$$

since $\displaystyle \lim \nolimits _{n \rightarrow \infty }\omega _t^{\nu _n,R,n} = \omega _t^R$ ${\mathbb {P}}$-almost surely in $L^2({\mathbb {T}}^2)$ (see Sect. 4), and $\displaystyle \sup \nolimits _{n>1}{\mathbb {E}}[\Vert a_n\Vert _{s,2}^4] < \infty$ by Lemma 25vi. and Proposition 17. $\square$

### Remark 28

i. In Lemma 27 we cannot follow an approach identical to the one used in Lemma 25 vi, due to a lack of smoothness in the truncated Eq. (11). This difficulty could be overcome if we embed $L^{2}\left( {\mathbb {T}}^{2}\right)$ into $L^{2}\left( {\mathbb {T}}^{2};{\mathbb {C}}\right)$, consider the basis of functions $\left\{ e^{2\pi i\xi \cdot x};\xi \in {\mathbb {Z}}^{2}\right\}$, and express $\omega _t^R$ as $\sum _{\xi \in {\mathbb {Z}}^{2} }\left| \xi \right| ^{2k}\left\langle \omega _t^R,\varphi _\xi \right\rangle \varphi _\xi .$ where $\varphi _\xi :{\mathbb {T}}^{2}\mapsto {\mathbb {C}}$, $\varphi _\xi (x)=e^{2\pi i\xi \cdot x}$, $x\in {\mathbb {T}}$ and $\xi \in {\mathbb {Z}}^{2}$. Then $\partial ^{k}\omega _t^R$ will be square integrable if and only if $\sum _{\xi \in {\mathbb {Z}}^{2} }\left| \xi \right| ^{2k}\left\langle \omega _t^R,\varphi _\xi \right\rangle ^{2}<\infty$ and we can finish the proof using the weak form of the truncated equation.
ii. Except the estimates which involve the second order operator , all the other estimates derived in Lemma 25 for $\omega ^{\nu _n,R,n}$ hold also for $\omega ^R$.

In what follows we recall some basic results which have been used before.

### Theorem 29

(Kurtz’s criterion for relative compactness—[23] Theorem 8.6) Let (*E*, *d*) be a complete and separable metric space, $(X^{\alpha })_{\alpha }$ a family of processes with càdlàg sample paths, and suppose that for every $\eta >0$ and any rational $t\ge 0$ there exists a compact set $K_{\eta , t} \subset E$ such that $\displaystyle \sup \nolimits _{\alpha }{\mathbb {P}}\big (X_{t}^{\alpha } \notin K_{\eta , t}\big ) \le \eta .$ Then the following two statements are equivalent:

- $(X^{\alpha })_{\alpha }$ is relatively compact.
- For each $T>0$ there exists $\beta >0$ and a family $(\gamma _{\delta }^{ \alpha })_{0<\delta <1, \ \hbox {all} \ \alpha }$ of nonnegative random variables such that ${\mathbb {E}} \big [{\tilde{d}}^\beta (X_{t}^{\alpha }, X_{t+u}^{\alpha }) | {\mathcal {F}}_t^{\alpha }\big ] \le {\mathbb {E}}\big [ \gamma _{\delta }^{\alpha } | {\mathcal {F}}_t^{\alpha } \big ]$ and $\displaystyle \lim \nolimits _{\delta \rightarrow 0} \sup \nolimits _{\alpha } {\mathbb {E}}\big [ \gamma _{\delta }^{\alpha } \big ] =0$ for $t \in [0, T], u \in [0, \delta ]$, where ${\tilde{d}}= d \wedge 1$ and the filtration $({\mathcal {F}}_t^{\alpha })_t$ refers to the natural filtration $({\mathcal {F}}_t^{X^{\alpha }})_t$.

### Theorem 30

(Gagliardo–Nirenberg [47]) Let $u \in L^{q}$ and $D^{m}u \in L^r$ with $1\le q, r\le \infty$. Then there exists a constant C such that the following inequalities hold for $D^{j}u$ with $0\le j<m$:

$$\begin{aligned} \Vert D^j u\Vert _p \le C \Vert D^mu\Vert _r^a\Vert u\Vert _q^{1-a} \end{aligned}$$

where $a \in [j/m, 1]$ is defined such that $\frac{1}{p} = \frac{j}{2} + a \big (\frac{1}{r}-\frac{m}{2}\big ) + \frac{1-a}{q}.$

### Remark 31

We denote by $(S^n(t))_t$ the semigroup of the generator $A:=\nu _n\Delta$. This semigroup is strongly continuous (see [43]) and for any $f \in L^2({\mathbb {T}}^2)$ it is true that

$$\begin{aligned} \Vert S^n(t)f\Vert _{k,2} \le \Vert f\Vert _{k,2}. \end{aligned}$$

### Theorem 32

(Theorem 4.2 in [41] or Theorem 1 in [34]) Let $({\mathcal {F}}_t^n)_t$ be a filtration and $(X^n)_n$ a sequence of $({\mathcal {F}}_t^n)_{t}$-adapted processes with càdlàg trajectories with values in a separable Hilbert space *H*. Let $(W^n)_n$ be a sequence of cylindrical Brownian motions on *H* (cf. Example 3.18 in [41]). If $(X^n, W^n)_n$ converges in distribution to (*X*, *W*), in the Skorokhod topology, then $(X^n, W^n, \int X^n dW^n)$ converges in distribution to $(X, W, \int XdW)$ in the Skorokhod topology. If the first convergence holds in probability, then the convergence of the stochastic integrals holds also in probability.

## Footnotes

1 In other words, we identify conditions under which the (strong) solution of the two-dimensional stochastic Euler equation with noise of transport type belongs to the Sobolev space ${\mathcal {W}}^{k,2}$ with *k* arbitrarily high.

2 Here *c* is a non-negative constant, $I_2$ is the identity matrix, and $\xi (x)^{\star }$ is the transpose of $\xi (x)$.

3 Here and later whenever the space *X* coincides with the Euclidean space ${\mathbb {R}}$ or ${\mathbb {R}}^2$, it is omitted from the notation: For example $L^{p} ({\mathbb {T}}^{2}; X)$ becomes $L^{p} ({\mathbb {T}}^{2})$, etc.

4 Here and everywhere else *u* is implicitly defined as the velocity field whose vorticity is $\omega$, in other words $\omega =curl\ u=\partial _2 u^1-\partial _1 u^2$. See further details in Remarks 4 and 21.

5 Equation (7) is interpreted as an identity between elements in $L^{2}({\mathbb {T}}^{2};{\mathbb {R}})$. The same applies to the identity (3)

6 We use the “check” notation in the description of the various components of a weak probabilistic solution, to emphasize that the existence of a weak solution does not guarantee that, for a *given* set of Brownian motions $(W^i)_i$ defined on a (possibly different) probability space $(\Omega , {\mathcal {F}}, {{\mathbb {P}}})$ a solution of (1) will exist. Clearly the existence of a strong solution implies the existence of a martingale solution.

7 These two extensions of Theorems 7 and 8 have been suggested to us by an anonymous referee. We thank the referee for this.

8 The application of the Lemma requires that the two solutions $\omega ^1$ and $\omega ^2$ belong to ${\mathcal {W}}^{k,2}({\mathbb {T}}^2)$ with $k\ge 2$. To deduce (9) we need a similar control (albeit not an identity) for higher order derivatives. This is done by using the approximating sequence constructed in Sect. 4 and then taking the limit. This is the reason why we cannot prove directly (9).

9 The operator $curl^{-1}$ is the convolution with the Biot–Savart kernel, see Remark 21 for details.

10 The stochastic Itô integral is understood here in the usual sense, see [20] .

11 Note that this implies continuity also for the original global solution i.e. $\omega \in C([0,T], {\mathcal {W}}^{k,2}({\mathbb {T}}^2)).$

12 In fact the paths are continuous in $L^2({\mathbb {T}}^2)$, however Kurtz’ criterion only requires càdlàg paths.

13 Since $t\rightarrow \Vert u_{t}^{n}\Vert _{k-1,\infty }$ is not uniformly bounded on $\left[ 0,T\right]$, a localisation argument is required here and the fact that ${\mathbb {E}}\left[ \sup \nolimits _{t\in \left[ 0,T\right] }\Vert u_{t}^{n}\Vert _{k-1,\infty }\right] <\infty .$

14 Kelvin’s conservation of circulation has been shown in [22].

15 We thank James-Michael Leahy for pointing out this argument to us.
